# Supplementary material for: Platelet-derived thrombospondin 1 promotes immune cell liver infiltration and exacerbates diet-induced steatohepatitis
Source: JHEP Rep. 2024 Jan 26;6(4):101019. doi: 10.1016/j.jhepr.2024.101019 (PMC10918562; doi:10.1016/j.jhepr.2024.101019)

Platelet-derived thrombospondin 1 promotes immune cell liver infiltration and exacerbates diet-induced steatohepatitis

Taesik Gwag, Sangderk Lee, Zhenyu Li, Alana Newcomb, Josephine Otuagomah, Steven A. Weinman, Ying Liang, Changcheng Zhou, Shuxia Wang

Table of contents

Supplementary materials and methods……………………………………………….……2

Supplementary references………………………………………………………,………,...4

Fig. S1………………………………………………………………………………,……5

Fig. S2………………………………………………………………………………,……6

Fig. S3………………………………………………………………………………,……7

Fig. S4………………………………………………………………………………,……8

Fig. S5………………………………………………………………………………,……9

Table S1…………………………………………………………………………...10

Table S2…………………………………………………………………………...12

Table S3…………………………………………………………………………...13

Whole western blot………………………………………………………………14

Supplementary materials and methods

Platelet preparation and aggregation

Blood was collected in ACD (85 mM trisodium citrate, 83 mM dextrose, and 21 mM citric acid) buffer. Platelets were isolated, washed, and resuspended in modified Tyrode's buffer (12 mM $NaHCO_3$, 138 mM NaCl, 5.5 mM glucose, 2.9 mM KCl, 2 mM $MgCl_2$, 0.42 mM NaH_2PO_4, 10 mM HEPES, pH 7.4). Platelet aggregation at 37 °C was measured by using a turbidimetric platelet aggregometer (Chrono-Log) with Collagen or Par4 (protease-activated receptor 4) agonist peptide (AYPGKF, from GenScript) stimulation as previously described (1, 2).

Mice bleeding time

Mice were anesthetized through inhalation of 2–5% isoflurane. Subsequently, the distal portion of the tail was cut and immersed in a warm saline solution. The time to stable cessation of bleeding was recorded as previous described (1).

Quantification of fat cell size and frequency distribution

H&E staining images from different fat depots' sections (eWAT, sWAT and BAT) were acquired. Adipocytes size was quantified by Image J and the frequency distribution was determined as previously described (3). Over 100 adipocytes were counted in each of 3 random sections per sample (n=5). We removed below size of 350 μm^2 from eWAT and sWAT and below size of 100 μm^2 from BAT to avoid including SVF (stromal vascular fraction) or non-adipocytes (4) (and a online eBook: Stock and Cinti, Encyclopedia of food sciences and Nutrition, 2003).

3T3-L1 (white preadipocyte cell line) and platelets co-culture, proliferation, and differentiation

Preadipocyte cell line, 3T3-L1 cells (ATCC), and wild-type or TSP1-deficient platelets ($4.5x10^4$/well, isolated from male 8-week-old mice) were co-cultured in a 24-well plate (with a

transwell insert) in growth medium (DMEM media with 5% FBS and 1% penicillin-streptomycin). The proliferation rate of 3T3-L1 cells was determined by a cell counter after 1, 3, 5, and 7 days of co-culture, and the growth curve was plotted. To determine whether platelet derived TSP1 affected 3T3 cell differentiation, confluent 3T3 cells were co-cultured with wild type or TSP1 deficient platelets (4.5×10^4/well) and treated with differentiation medium containing 0.5 mM IBMX, 1 μM dexamethasone, and 1 μM insulin in the growth media for 4-5 days (considering the short survival time of platelets in *in vitro* conditions). Lipid accumulation in differentiated adipocytes was determined by Oil red O staining. Expression of white adipocyte cell markers (AP2, C/EBPa, C/EBPb, PPAR-r, and adiponectin) were determined by qPCR.

T37i (brown preadipocyte cell line) differentiation, platelets co-culture, and Nrg4 production

T37i cells (provided by Dr. Jun Liu from Mayo Clinic) were cultured in growth medium (DMEM/F-12 containing 10% FBS, 1% penicillin-streptomycin, and 20 mM HEPES) in a 24-well plate. When cells reached full confluence, differentiation was induced by adding differentiation medium (growth medium containing 45 nM insulin and 10 μM T4) for 7 days. After differentiation, cells were cultured with wild-type or TSP1-deficient platelets (4.5x104/well) for 3 days. The Nrg4 or other gene expression level in cells was determined by qPCR, and the Nrg4 level in the conditioned medium was measured using the Nrg4 ELISA kit (Novus Biologicals, CO, USA).

Immunofluorescence staining of fat tissue with anti-CD41 antibody (maker for platelets)

To investigate platelet infiltration into fat tissues, paraffin sections from eWAT, sWAT, and BAT were stained with anti-CD41-PE (1:100, Thermofisher) overnight at 4°C. DAPI was used to stain nuclei. Images were captured by a Nikon Eclipse 55i microscope. Semi-quantification of CD41+ cells in liver sections was performed as previously described (5).

Statistical analysis

Statistical analysis was performed using Prism version 9.0 (GraphPad Software, San Diego, CA, USA). Data are expressed as mean values ± SE. Two-tailed Student's t-test was used to determine statistical significance between the two groups. One-way ANOVA followed by Tukey's multiple comparison test or 2-way ANOVA followed by Tukey's multiple comparison test was used for multi-group comparisons.

Supplementary references

1. Xiang B, Zhang G, Ye S, Zhang R, Huang C, Liu J, Tao M, et al. Characterization of a Novel Integrin Binding Protein, VPS33B, Which Is Important for Platelet Activation and In Vivo Thrombosis and Hemostasis. Circulation 2015;132:2334-2344.
2. Lu DH, Hsu CC, Huang SW, Tu HJ, Huang TF, Liou HC, Liao HM, et al. ARHGEF10 knockout inhibits platelet aggregation and protects mice from thrombus formation. J Thromb Haemost 2017;15:2053-2064.
3. Parlee SD, Lentz SI, Mori H, MacDougald OA. Quantifying size and number of adipocytes in adipose tissue. Methods Enzymol 2014;537:93-122.
4. Palomäki VA, Koivukangas V, Meriläinen S, Lehenkari P, Karttunen TJ. A Straightforward Method for Adipocyte Size and Count Analysis Using Open-source Software QuPath. Adipocyte 2022;11:99-107.
5. Gwag T, Ma E, Zhou C, Wang S. Anti-CD47 antibody treatment attenuates liver inflammation and fibrosis in experimental non-alcoholic steatohepatitis models. Liver Int 2022;42:829-841.

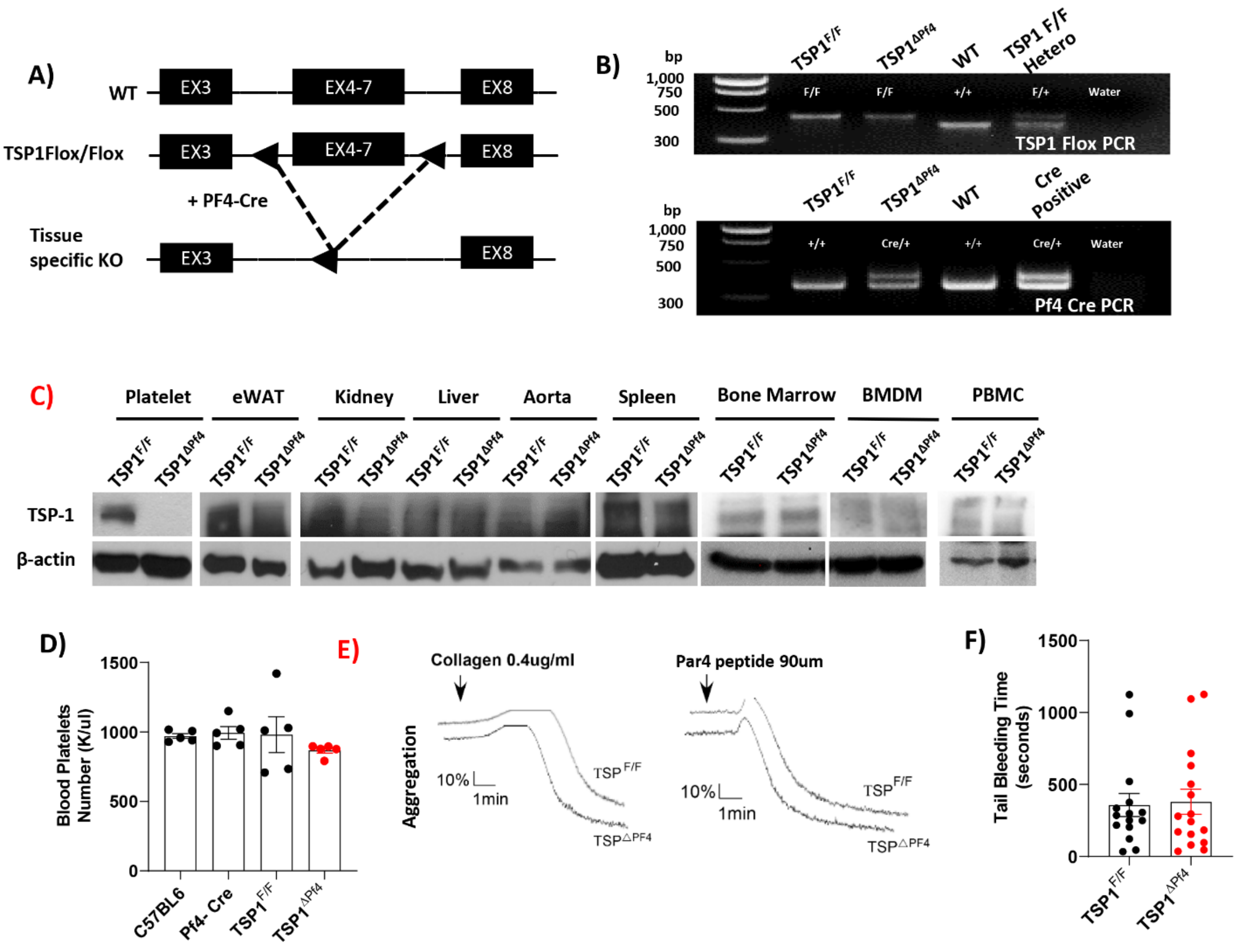

Fig. S1: Generation and characterization of mice with platelet specific deletion of TSP1 ($TSP1^{\Delta pf4}$).

A). Strategy for generation of $TSP1^{\Delta pf4}$ mice by breeding $TSP1^{F/F}$ mice with PF4-Cre mice; B) PCR analysis of genomic DNA from tail of wild type (WT), heterozygous, $TSP1^{F/F}$, or $TSP1^{\Delta pf4}$ mice from 8 week old male mice for detection of floxed gene and Cre gene, respectively; C) Expression of TSP1 from isolated platelets, various tissues or blood immune cells from 8 weeks old male mice was determined by immunoblotting; D) Blood platelet number; E) Platelet aggregation measured in a lumi-Aggregometer with Collagen or Par4 peptide stimulation and F) Mice tail bleeding time were measured. Results are mean ± SE (n=5 or 16 mice/group). eWAT: white adipose tissue (from epididymal fat); PLT: platelet; BMDM: bone marrow derived macrophages; PBMC: peripheral blood mononuclear cells; Par4: protease-activated receptor 4

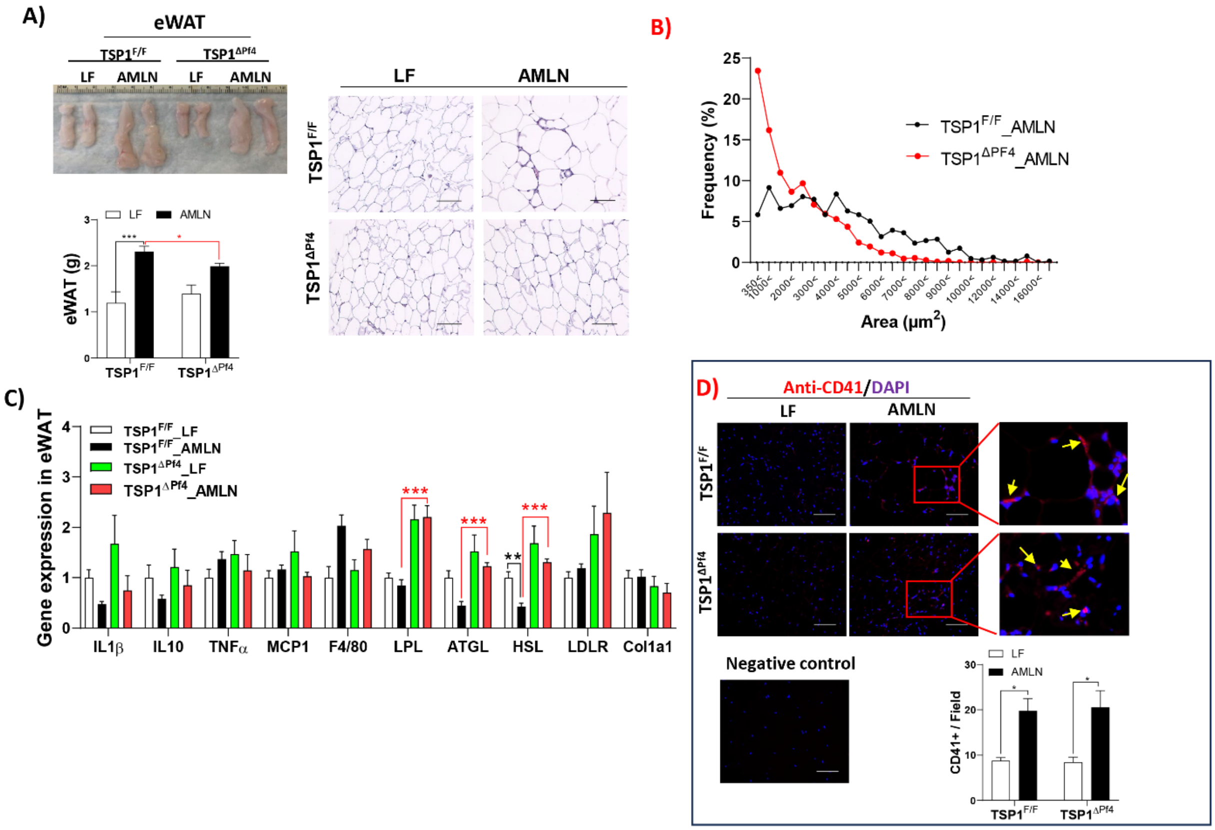

Fig. S2: Changes in epididymal white fat tissue from TSP1$^{\Delta pf4}$ mice after 32 weeks of AMLN diet feeding

(A). Weight and representative H&E staining images of epididymal fat (eWAT) from 32 weeks of LF or AMLN fed TSP1 $^{F/F}$ mice or TSP1$^{\Delta pf4}$ mice; (B). The distribution of adipocyte sizes; (C) qPCR of gene expression; Data are represented as mean ± SE (n=3-5 mice/group); 2-way ANOVA with Tukey's multiple comparisons test. *P<0.05 and *** P<0.001; (D). Representative fluorescence images of eWAT staining with anti-CD41 antibody (a platelet marker). Positive staining was shown as yellow arrows.

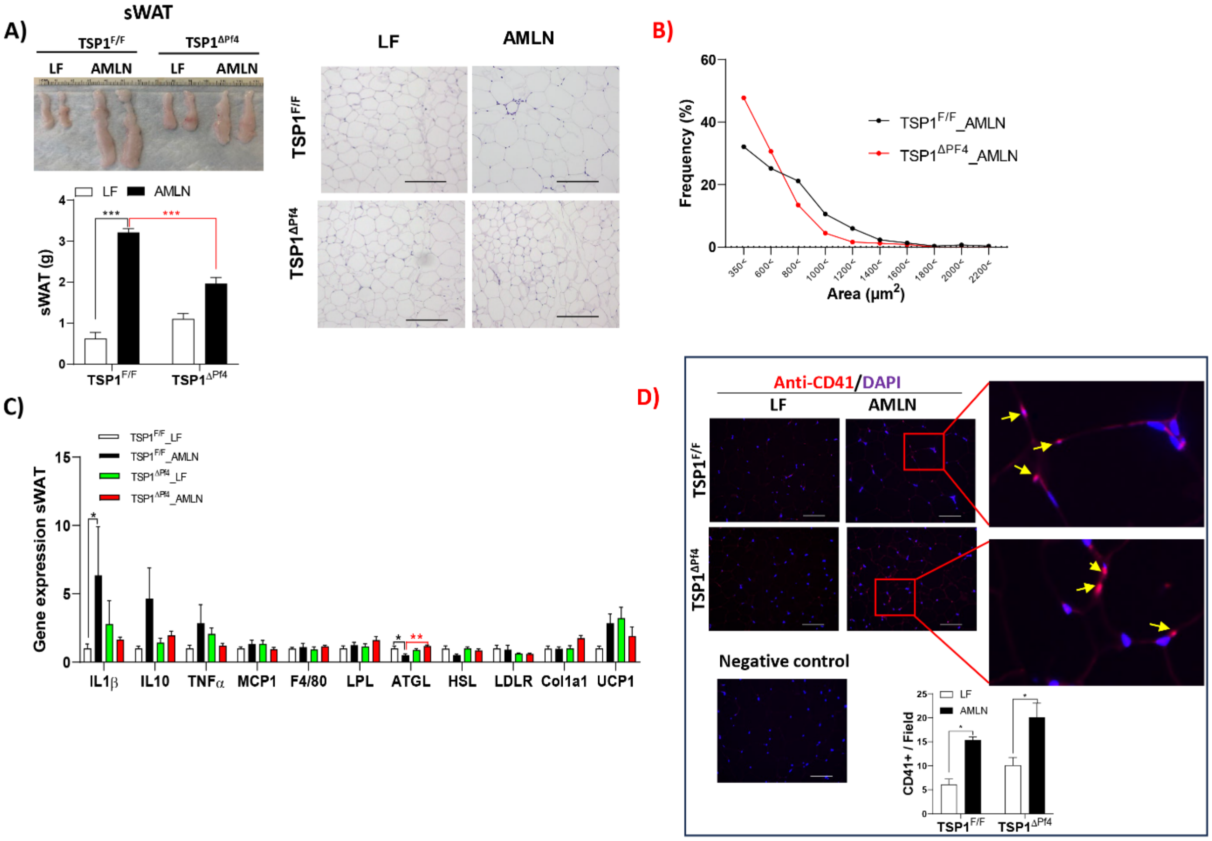

Fig. S3: Changes in subcutaneous white fat tissue from TSP1$^{\Delta pf4}$ mice after 32 weeks of AMLN diet feeding

(A). Weight and representative H&E staining images of subcutaneous fat (sWAT) from 32 weeks of LF or AMLN fed TSP1$^{F/F}$ mice or TSP1$^{\Delta pf4}$ mice; (B). The distribution of adipocyte sizes; (C) qPCR of gene expression. Data are represented as mean ± SE (n=3-5 mice/group); 2-way ANOVA with Tukey's multiple comparisons test. *P<0.05, ** P<0.01 and *** P<0.001; (D). Representative fluorescence images of sWAT staining with anti-CD41 antibody (a platelet marker). Positive staining was shown as yellow arrows.

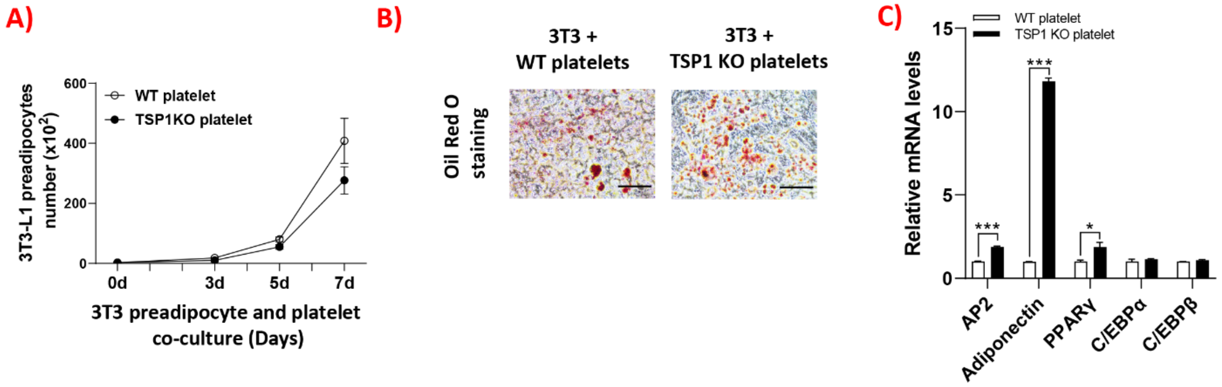

Fig. S4: Co-culture of platelets with 3T3-L1 preadipocytes on 3T3 preadipocyte proliferation and differentiation

(A). Proliferation curve of 3T3-L1 preadipocytes after various days of co-culturing with WT or TSP1KO platelets. Differentiation was induced in co-cultured 3T3-L1 cell and platelets. Representative Oil Red O staining (B) and qPCR of gene expression in differentiated 3T3 cells (C) were performed. Data are represented as mean ± SE (n=3); 2-way ANOVA with Tukey's multiple comparisons test. *P<0.05 and *** P<0.001

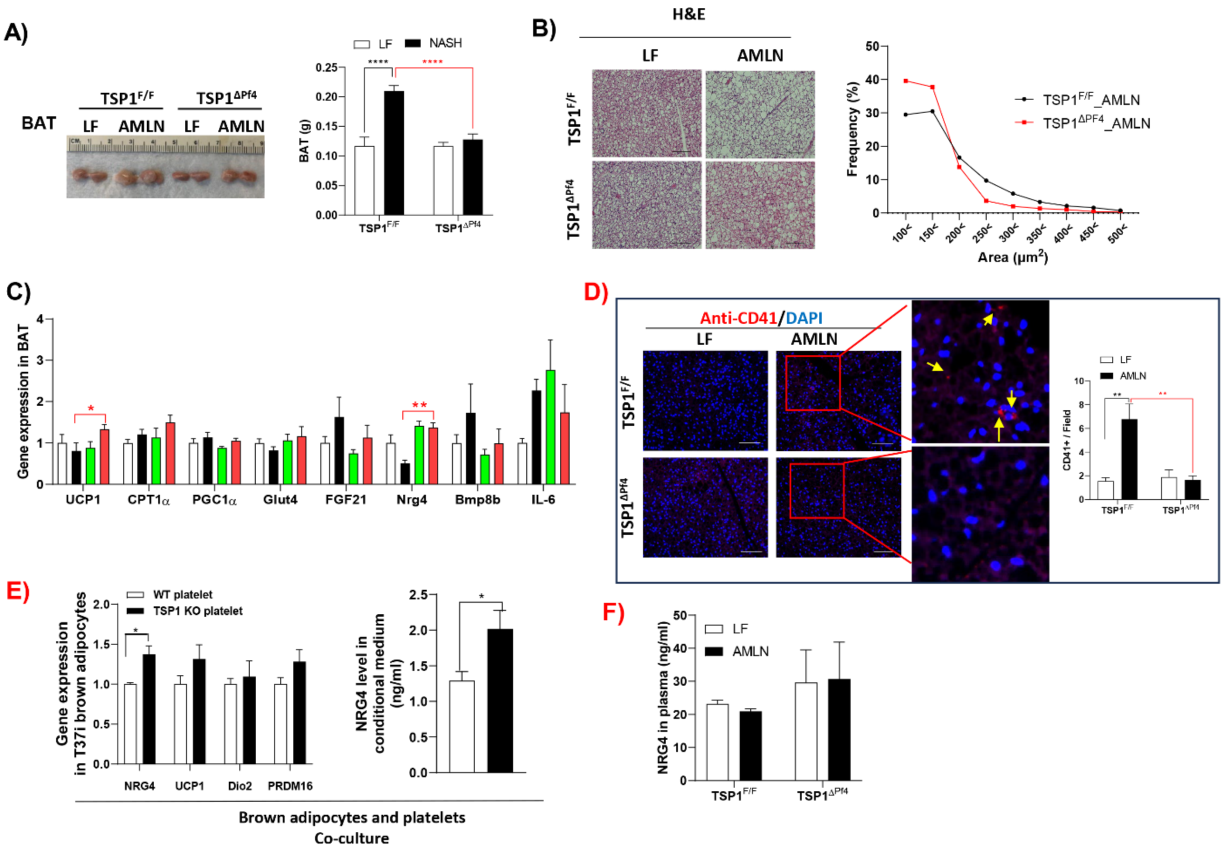

Fig. S5: Changes in brown fat tissue from *TSP1$^{\Delta pf4}$* mice after 32 weeks of AMLN diet feeding and in vitro platelets and brown adipocytes co-culture

(A). Weight and representative H&E staining images of BAT from 32 weeks of LF or AMLN fed TSP1$^{F/F}$ mice or TSP1$^{\Delta pf4}$ mice; (B). The distribution of adipocyte sizes; (C) qPCR of gene expression; (D). Representative fluorescence images of BAT staining with anti-CD41 antibody (a platelet marker). Positive staining was shown as yellow arrows; (E). qPCR of gene expression or Nrg4 protein levels in condition media in T37i brown adipocytes after co-cultured with WT or TSP1KO platelets for 3 days (n=3 experiments); (F). Plasma Nrg4 protein levels from 32 weeks of LF or AMLN fed *TSP1$^{F/F}$* mice or *TSP1$^{\Delta pf4}$* mice. Data are represented as mean ± SE (n=3-5 mice/group); 2-way ANOVA with Tukey's multiple comparisons test. *P<0.05, ** P<0.01 and **** P<0.0001

Table S1. Primer Sequences for qPCR

Gene	Primer sequence	Genes	Primer sequence
**Mouse primers**			
CPT1α	5'-CTCTATGTGGTGTCCAAG-3'<br>5'-CACAGGACACATAGTCAG-3'	PGC1α	5'-CTGCATGAGTGTGTGCTGTG-3'<br>5'-CAAATATGTTCGCAGGCTCA-3'
Glut4	5'-CATGGCTGTCGCTGGTTTC-3'<br>5'-AAACCCATGCCGACAATGA-3'	FGF21	5'-GCTGCTGGAGGACGGTTACA-3'<br>5'-CACAGGTCCCCAGGATGTTG-3'
Nrg4	5'-ATGCCAACAGATCACGAGC-3'<br>5'-TCTTCAGTGTTCTCTGTGGCTG-3'	Bmp8b	5'-CAACCACGCCACTATGCAG-3'<br>5'-CACTCAGCTCAGTAGGCACA-3'
F4/80	5'-CTTTGGCTATGGGCTTCCAGTC-3'<br>5'-GCAAGGAGGACAGAGTTTATCGTG-3'	CD11b	5'-CGGAAAGTAGTGAGAGAACTGTTTC-3'<br>5'-TTATAATCCAAGGGATCACCGAATTT-3'
UCP1	5'-ACTGCCACACCTCCAGTCATT-3'<br>5'-CTTTGCCTCACTCAGGATTGG-3'	MCP-1<br>(CCl2)	5'-CAGCCAGATGCAGTTAACGC-3'<br>5'-GCCTACTCATTGGGATCATCTTG-3'
IL-1β	5'-TGGAGAGTGTGGATCCCAAGCAAT-3'<br>5'-TGTCCTGACCACTGTTGTTTCCCA-3'	TNFα	5'-AGCCGATGGGTTGTACCT-3'<br>5'-TGAGTTGGTCCCCCTTCT-3'
TGFβ	5'-ACAATTCCTGGCGTTACC-3'<br>5'-GGCTGATCCCGTTGATTT-3'	α-SMA	5'-ATTGTGCTGGACTCTGGAGATGGT-3'<br>5'-TGAGTCACGGACAATCTCACGCT-3'
TIMP1	5'-TCTTGGTTCCCTGGCGTACTCT-3'<br>5'-GTGAGTGTCACTCTCCAGTTTGC-3'	Col1a1	5'-TTCTCCTGGCAAAGACGGACTCAA-3'<br>5'-AGGAAGCTGAAGTCATAACCGCCA-3'
IL-6	5'-TGGCTAAGGACCAAGACCATCCAA-3'<br>5'-AACGCACTAGGTTTGCCGAGTAGA-3'	CD68	5'- CAAGGTCCAGGGAGGTTGTG -3'<br>5'- CCAAAGGTAAGCTGTCCATAAGGA -3'
Clec4f	5'-CTTCGGGGAAGCAACAACTC-3'<br>5'-CAAGCAACTGCACCAGAGAAC-3'	CXCL1	5'- TAGTAGAAGGGTGTTGTG-3'<br>5'- GTAACAGTCCTTTGAACG-3'
CXCL2	5'-CTG TCT GAG AGT TCA CTT A-3'<br>5'-GTA GCT AGT TCC CAA CTC-3'	CXCL4	5'- CCCTAGACCCATTTCCTCAA-3'<br>5'- AGAAACAACAGGCCCAGAAG-3'
CXCL5	5'-ACAGTGCCCTACGGTGGAAGT-3'<br>5'-CGAGTGCATTCCGCTTAGCTT-3'	CXCL7	5'-GGAAAATCTGATGGCATGGAC-3'<br>5'-CAGGCACGTTTTTTGTCCATTCT-3'
CXCL10	5'-CCTCATCCTGCTGGGTCTG-3'<br>5'-CTCAACACGTGGGCAGGA-3'	CXCL12	5'-TGCATCAGTGACGGTAAACCA-3'<br>5'-AGATGCTTGACGTTGGCTCT-3'
CXCR2	5'-TCACAAACAGCGTCGTAGA-3'<br>5'-GACAGCATCTGGCAGAATAG-3'	CXCR4	5'-TCAGTGGCTGACCTCCTCTT-3'<br>5'-CTTGGCCTTTGACTGTTGGT-3'

IL10	5'- GCTCTTACTGACTGGCATGAG- 3'<br>5'- CGCAGCTCTAGGAGCATGTG -3'	CCR2	5’-AGAGAGCTGCAGCAAAAAGG-3’<br>5’-GGAAAGAGGCAGTTGCAAAG-3’
ATGL	5'- AACACCAGCATCCAGTTCAA-3'<br>5'- GGTTCAGTAGGCCATTCCTC-3'	LPL	5’-GGGAGTTTGGCTCCAGAGTTT-3’<br>5’-TGTGTCTTCAGGGGTCCTTAG-3’
LDLR	5'-GCTCCATAGGCTATCTGCTCTTCA-3'<br>5'-GCGGTCCAGGGTCATCTTC-3'	HSL	5’-GGCTCACAGTTACCATCTCACC-3’<br>5’-GAGTACCTTGCTGTCCTGTCC-3’
AP2	5'-AAGCCCACTCCCACTTCTTT-3'<br>5'-TCACCTGGAAGACAGCTCCT-3'	Dio2	5'-CATCTTCCTCCTAGATGCCTA-3'<br>5'-CTGATTCAGGATTGGAGACGTG-3'
Adiponectin	5'-AACATTCCGGGACTCTACT-3'<br>5'-TACTGGTCGTAGGTGAAGAG-3'	PRDM16	5'-CACAAGACATCTGAGGACAC-3'<br>5'-CTCGTGTTCGTGCTTCTT-3'
PPARγ	5'-TGCTGTTATGGGTGAAACTCTG-3'<br>5'-CTGTGTCAACCATGGTAATTTCTT-3'	C/EBPα	5'-TGCCTCCCAGAGGACCAATGAAAT-3'<br>5'-TGTGTGTATGAACTGGCTGGAGGT-3'
C/EBPβ	5'-TGATGCAATCCGGATCAAACGTGG-3'<br>5'-TTTAAGTGATTACTCAGGGCCCGGCT-3'		
**Human primers**			
α-SMA	5’-CCAGAGCCATTGTCACACAC-3’<br>5’-CAGCCAAGCACTGTCAGG-3’	IL-1β	5’-CAACAGGCTGCTCTGGGATT-3’<br>5’-CATGGCCACAACAACTGACG-3’
TIMP	5’-GGAGAGTGTCTGCGGATACTTC-3’<br>5’-GCAGGTAGTGATGTGCAAGAGTC-3’	β-Actin	5’-CATGTACGTTGCTATCCAGGC -3’<br>5’-CTCCTTAATGTCACGCACGAT -3’

Table S2. Blood profile for 8 weeks-old mice

Parameter	$TSP^{F/F}$	$TSP1^{\Delta pf4}$
WBC count (k/ul)	4.24±0.754	4.24±0.545
Neutrophil count (K/ul)	0.73±0.245	1.11±0.591
Lymphocytes count (K/ul)	3.36±0.529	2.89±0.666
Monocytes count (K/ul)	0.06±0.024	0.05±0.025
Eosinophil count (K/ul)	0.09±0.027	0.05±0.025
RBC (M/ul)	9.59±0.469	9.20±0.173
Hemoglobin (g/dL)	14.32±0.602	13.78±0.286
MCV (fL)	50.68±0.712	50.84±0.631
MCH (Pg)	14.94±0.195	14.98±0.311
MCHC (g/dL)	29.48±0.402	29.44±0.241
RDW (%)	23.36±0.404	22.24±0.522
Platelet (K/ul)	980.4±287.863	868±43.537

Abbreviations: RBC: red blood cell; MCV: mean corpuscular volume; MCH: Mean corpuscular hemoglobin; MCHC: mean corpuscular hemoglobin concentration; RDW: red cell distribution width; MPV: mean platelet volume

Table S3. Blood profile in mice after 32 weeks AMLN or LF diet feeding

Parameter	$TSP1^{F/F}$_LF	$TSP1^{F/F}$_AMLN	$TSP1^{\Delta pf4}$_LF	$TSP1^{\Delta pf4}$_AMLN
WBC count (k/ul)	5.26±1.265	8.19±3.875 *	5.25±1.911	4.95±1.088 ##
Neutrophil count (K/ul)	1.99±1.157	1.53±0.402	1.93±1.187	1.75±0.958
Lymphocytes count (K/ul)	3.12±1.191	6.20±3.618 ***	3.02±1.512	3.04±1.324 ###
Monocytes count (K/ul)	0.09±0.072	0.23±0.075 **	0.07±0.036	0.13±0.194 ##
Eosinophil count (K/ul)	0.08±0.049	0.14±0.061	0.09±0.046	0.10±0.050
RBC (M/ul)	10.02±0.438	10.28±1.563	10.04±0.707	9.95±0.995
Hemoglobin (g/dL)	14.74±0.872	14.60±2.250	14.49±1.138	14.27±1.223
MCV (fL)	49.50±1.920	47.89±1.176 *	48.17±1.040	47.74±0.632
MCH (Pg)	14.64±0.346	14.19±0.398*	14.43±0.369	14.30±0.286
MCHC (g/dL)	29.84±0.536	29.65±0.655	29.95±0.538	30.12±0.960
RDW (%)	25.22±1.364	26.03±1.584	29.61±1.189	30.63±2.302
Platelet (K/ul)	987±422.570	1097±146.007	1254±153.848	1258±262.188

Note: ****P*<0.05, *******P*<0.01, and ********P*<0.001 as compared to $TSP1^{F/F}$_LF;

#*P*<0.05 and ## *P*<0.01 as compared to $TSP1^{F/F}$_AMLN

Abbreviations: RBC: red blood cell; MCV: mean corpuscular volume; MCH: Mean corpuscular hemoglobin; MCHC: mean corpuscular hemoglobin concentration; RDW: red cell distribution width; MPV: mean platelet volume

Whole western blot for Figure 3G, Figure 4B

Fig. 3G

α-SMA in liver

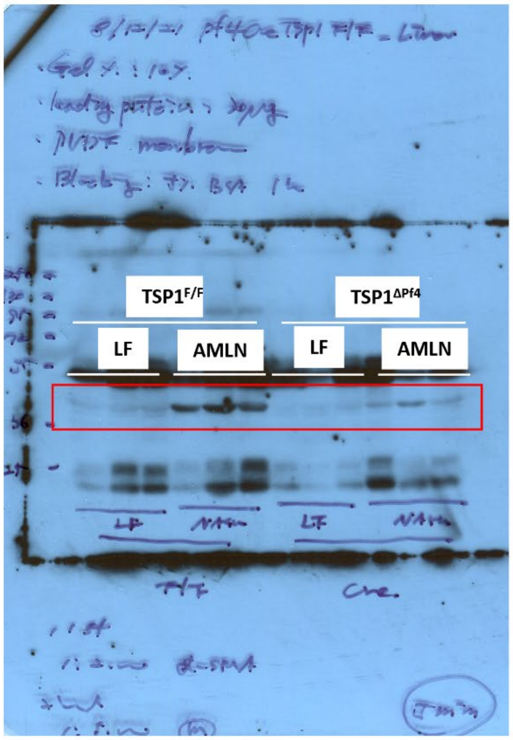

β-actin for α-SMA

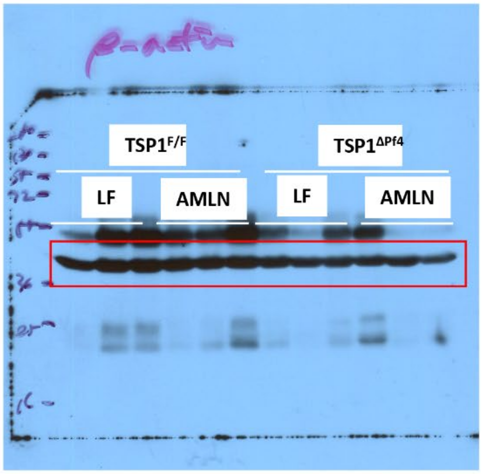

Fig4. B

TSP1 in liver (reducing gel)

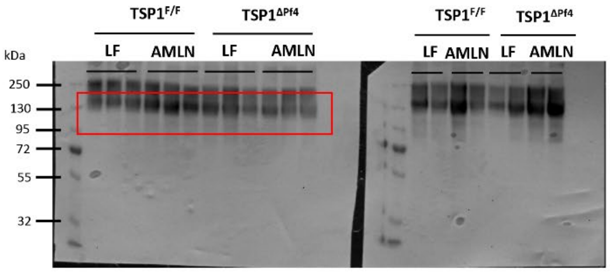

β-actin for TSP1 in liver

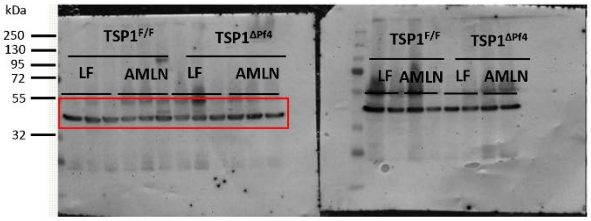

Whole western blot for Figure S1

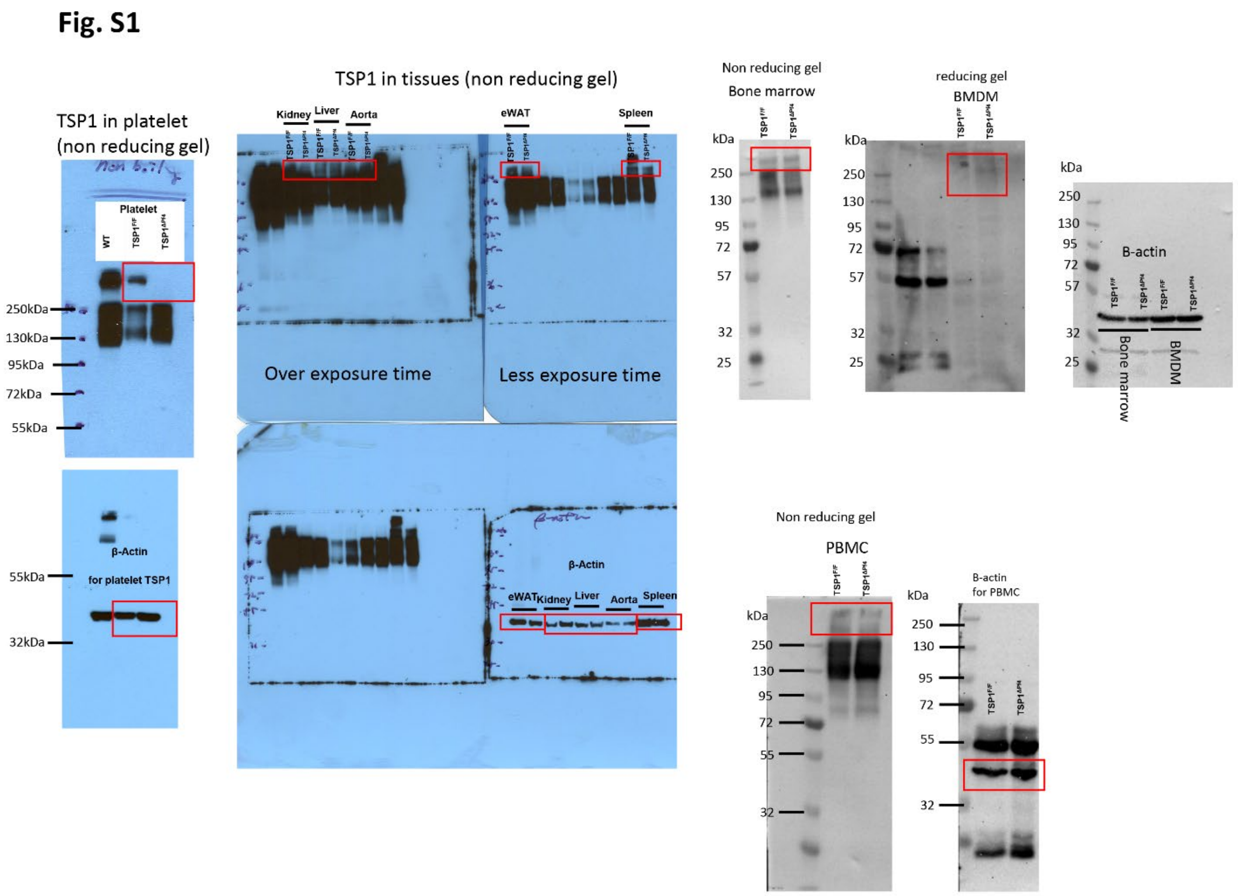

Supplement: Multimedia component 1 [file mmc1.pdf]
