# Supplementary material for: Platelet-derived thrombospondin 1 promotes immune cell liver infiltration and exacerbates diet-induced steatohepatitis
Source: JHEP Rep. 2024 Jan 26;6(4):101019. doi: 10.1016/j.jhepr.2024.101019 (PMC10918562; doi:10.1016/j.jhepr.2024.101019)
Supplement: Multimedia component 2 [file mmc2.docx]

**JHEP Reports**

**CTAT methods**

Tables for a “Complete, Transparent, Accurate and Timely account” (CTAT) are now mandatory for all revised submissions. The aim is to enhance the reproducibility of methods.

- Only include the parts relevant to your study
- Refer to the CTAT in the main text as ‘Supplementary CTAT Table’
- Do not add subheadings
- Add as many rows as needed to include all information
- Only include one item per row

**If the CTAT form is not relevant to your study, please outline the reasons why:**

|  |
| --- |

- 1. **Antibodies**

| **Name** | **Citation** | **Supplier** | **Cat no.** | **Clone no.** |
| --- | --- | --- | --- | --- |
| **Anti-CD41** |  | **Thermofisher** | **PA5-22307** |  |
| **Anti-Neutrophil** |  | **Cedarlane** | **CL8993AP** | **7/4** |
| **Anti-CD4** | Muranushi H  et al., Blood Adv 7:106-121 (2023). | **Abcam** | **ab183685** | **SK3** |
| **Anti-CD8** | [Hoover AA, Hufnagel DH, Harris W et al., BMC Cancer 2020-09-27 [PMID: 33028251]](http://www.ncbi.nlm.nih.gov/pubmed/33028251) | **Novus bio.** | **NBP1-49045** | **53-6.7** |
| **Anti-TSP1** | Zhang X, Yang L, Szeto P et al. The Hippo pathway oncoprotein YAP promotes melanoma cell invasion and spontaneous metastasis bioRxiv (WB) | **Novus bio.** | **NB100-2059** | **A6.1** |
| **Anti-α-SMA** |  | **Sigma** | **A5228** | **1A4** |
| **Anti-β-Actin** |  | **Sigma** | **A5441** | **AC-15** |
| **Anti-CD41-PE** | Wong J, et al. 2015. Elife. 3: 07839. [PubMed](http://www.ncbi.nlm.nih.gov/pubmed/26193121) | **Biolegend** | **133905** | **MWReg30** |
| **Anti-p-selectin** | [Bondareva O, RodrIguez-Aguilera JR, Oliveira F et al. Nature metabolism 2022-11-01 [PMID: 36400935]](http://www.ncbi.nlm.nih.gov/pubmed/36400935) | **Novus bio.** | **NB100-65392SS** | **Psel.KO.2.7** |
| **Anti-mouse-Alexa488** |  | **Invitrogen** | **A11001** |  |
|  |  |  |  |  |

- 1. **Cell lines**

| **Name** | **Citation** | **Supplier** | **Cat no.** | **Passage no.** | **Authentication test method** |
| --- | --- | --- | --- | --- | --- |
| **HepG2** |  | **ATCC** |  | **P15** |  |
| **THP1** |  | **ATCC** |  | **P10** |  |
| **Human stellate cell** |  | **Zenbio** |  | **P5** |  |
| **T37i** |  | Provided by Dr. Jun Liu from Mayo Clinic |  | **P8** |  |
| **3T3-L1** |  | **ATCC** |  | **P10** |  |

- 1. **Organisms**

| **Name** | **Citation** | **Supplier** | **Strain** | **Sex** | **Age** | **Overall n number** |
| --- | --- | --- | --- | --- | --- | --- |
| **TSP1F/F** | **Memetimin et al PMID: 30351986 ; Taesik et al. PMID: 33294831** | **Generated in the lab** | **C57/B6** | **Male** | **8wks** | **34** |
| **Pf4-Cre/+** |  | **Jackson lab** | **C57/B6** | **Male** | **8wks** | **10** |
| **TSP1ΔPF4** |  | **Generated in the lab** | **C57/B6** | **Male** | **8wks** | **34** |

- 1. **Sequence based reagents**

| **Name** | **Sequence** | **Supplier** |
| --- | --- | --- |
| CPT1α | 5’-CTCTATGTGGTGTCCAAG-3’  5’-CACAGGACACATAGTCAG-3’ | IDT integrated DNA technologies |
| Glut4 | 5’-CATGGCTGTCGCTGGTTTC-3’  5’-AAACCCATGCCGACAATGA-3’ | IDT integrated DNA technologies |
| Nrg4 | 5’-ATGCCAACAGATCACGAGC-3’  5’-TCTTCAGTGTTCTCTGTGGCTG-3’ | IDT integrated DNA technologies |
| F4/80 | 5’-CTTTGGCTATGGGCTTCCAGTC-3’  5’-GCAAGGAGGACAGAGTTTATCGTG-3’ | IDT integrated DNA technologies |
| UCP1 | 5’-ACTGCCACACCTCCAGTCATT-3’  5’-CTTTGCCTCACTCAGGATTGG-3’ | IDT integrated DNA technologies |
| IL-1β | 5’-TGGAGAGTGTGGATCCCAAGCAAT-3’  5’-TGTCCTGACCACTGTTGTTTCCCA-3’ | IDT integrated DNA technologies |
| TGFβ | 5’-ACAATTCCTGGCGTTACC-3’  5’-GGCTGATCCCGTTGATTT-3’ | IDT integrated DNA technologies |
| TIMP1 | 5’-TCTTGGTTCCCTGGCGTACTCT-3’  5’-GTGAGTGTCACTCTCCAGTTTGC-3’ | IDT integrated DNA technologies |
| IL-6 | 5’-TGGCTAAGGACCAAGACCATCCAA-3’  5’-AACGCACTAGGTTTGCCGAGTAGA-3’ | IDT integrated DNA technologies |
| Clec4f | 5’-CTTCGGGGAAGCAACAACTC-3’  5’-CAAGCAACTGCACCAGAGAAC-3’ | IDT integrated DNA technologies |
| CXCL2 | 5’-CTG TCT GAG AGT TCA CTT A-3’  5’-GTA GCT AGT TCC CAA CTC-3’ | IDT integrated DNA technologies |
| CXCL5 | 5’-ACAGTGCCCTACGGTGGAAGT-3’  5’-CGAGTGCATTCCGCTTAGCTT-3’ | IDT integrated DNA technologies |
| CXCL10 | 5’-CCTCATCCTGCTGGGTCTG-3’  5’-CTCAACACGTGGGCAGGA-3’ | IDT integrated DNA technologies |
| CXCR2 | 5’-TCACAAACAGCGTCGTAGA-3’  5’-GACAGCATCTGGCAGAATAG-3’ | IDT integrated DNA technologies |
| IL10 | 5'- GCTCTTACTGACTGGCATGAG- 3'  5'- CGCAGCTCTAGGAGCATGTG -3' | IDT integrated DNA technologies |
| PGC1α | 5’-CTGCATGAGTGTGTGCTGTG-3’  5’-CAAATATGTTCGCAGGCTCA-3’ | IDT integrated DNA technologies |
| FGF21 | 5’-GCTGCTGGAGGACGGTTACA-3’  5’-CACAGGTCCCCAGGATGTTG-3’ | IDT integrated DNA technologies |
| Bmp8b | 5’-CAACCACGCCACTATGCAG-3’  5’-CACTCAGCTCAGTAGGCACA-3’ | IDT integrated DNA technologies |
| CD11b | 5’-CGGAAAGTAGTGAGAGAACTGTTTC-3’  5’-TTATAATCCAAGGGATCACCGAATTT-3’ | IDT integrated DNA technologies |
| MCP-1 (CCl2) | 5’-CAGCCAGATGCAGTTAACGC-3’  5’-GCCTACTCATTGGGATCATCTTG-3’ | IDT integrated DNA technologies |
| TNFα | 5’-AGCCGATGGGTTGTACCT-3’  5’-TGAGTTGGTCCCCCTTCT-3’ | IDT integrated DNA technologies |
| α-SMA | 5’-ATTGTGCTGGACTCTGGAGATGGT-3’  5’-TGAGTCACGGACAATCTCACGCT-3’ | IDT integrated DNA technologies |
| Col1a1 | 5’-TTCTCCTGGCAAAGACGGACTCAA-3’  5’-AGGAAGCTGAAGTCATAACCGCCA-3’ | IDT integrated DNA technologies |
| CD68 | 5'- CAAGGTCCAGGGAGGTTGTG -3'  5'- CCAAAGGTAAGCTGTCCATAAGGA -3' | IDT integrated DNA technologies |
| CXCL1 | 5'- TAGTAGAAGGGTGTTGTG-3'  5'- GTAACAGTCCTTTGAACG-3' | IDT integrated DNA technologies |
| CXCL4 | 5'- CCCTAGACCCATTTCCTCAA-3'  5'- AGAAACAACAGGCCCAGAAG-3' | IDT integrated DNA technologies |
| CXCL7 | 5’-GGAAAATCTGATGGCATGGAC-3’  5’-CAGGCACGTTTTTTGTCCATTCT-3’ | IDT integrated DNA technologies |
| CXCL12 | 5’-TGCATCAGTGACGGTAAACCA-3’  5’-AGATGCTTGACGTTGGCTCT-3’ | IDT integrated DNA technologies |
| CXCR4 | 5’-TCAGTGGCTGACCTCCTCTT-3’  5’-CTTGGCCTTTGACTGTTGGT-3’ | IDT integrated DNA technologies |
| CCR2 | 5’-AGAGAGCTGCAGCAAAAAGG-3’  5’-GGAAAGAGGCAGTTGCAAAG-3’ | IDT integrated DNA technologies |
| ATGL | 5'- AACACCAGCATCCAGTTCAA-3'  5'- GGTTCAGTAGGCCATTCCTC-3' | IDT integrated DNA technologies |
| LDLR | 5'-GCTCCATAGGCTATCTGCTCTTCA-3'  5'-GCGGTCCAGGGTCATCTTC-3' | IDT integrated DNA technologies |
| LPL | 5’-GGGAGTTTGGCTCCAGAGTTT-3’  5’-TGTGTCTTCAGGGGTCCTTAG-3’ | IDT integrated DNA technologies |
| HSL | 5’-GGCTCACAGTTACCATCTCACC-3’  5’-GAGTACCTTGCTGTCCTGTCC-3’ | IDT integrated DNA technologies |
| AP2 | 5'-AAGCCCACTCCCACTTCTTT-3'  5'-TCACCTGGAAGACAGCTCCT-3' | IDT integrated DNA technologies |
| Adiponectin | 5'-AACATTCCGGGACTCTACT-3'  5'-TACTGGTCGTAGGTGAAGAG-3' | IDT integrated DNA technologies |
| PPARγ | 5'-TGCTGTTATGGGTGAAACTCTG-3'  5'-CTGTGTCAACCATGGTAATTTCTT-3' | IDT integrated DNA technologies |
| Dio2 | 5'-CATCTTCCTCCTAGATGCCTA-3'  5'-CTGATTCAGGATTGGAGACGTG-3' | IDT integrated DNA technologies |
| PRDM16 | 5'-CACAAGACATCTGAGGACAC-3'  5'-CTCGTGTTCGTGCTTCTT-3' | IDT integrated DNA technologies |
| C/EBPα | 5'-CGCAAGAGCCGAGATAAAGC-3'  5'-CGGTCATTGTCACTGGTCAACT-3' | IDT integrated DNA technologies |
| C/EBPβ | 5'-TGATGCAATCCGGATCAAACGTGG-3'  5'-TTTAAGTGATTACTCAGGGCCCGGCT-3' | IDT integrated DNA technologies |
| α-SMA (human) | 5’-CCAGAGCCATTGTCACACAC-3’  5’-CAGCCAAGCACTGTCAGG-3’ | IDT integrated DNA technologies |
| TIMP (human) | 5’-GGAGAGTGTCTGCGGATACTTC-3’  5’-GCAGGTAGTGATGTGCAAGAGTC-3’ | IDT integrated DNA technologies |
| IL-1β (human) | 5’-CAACAGGCTGCTCTGGGATT-3’  5’-CATGGCCACAACAACTGACG-3’ | IDT integrated DNA technologies |
| β-Actin (human) | 5’-CATGTACGTTGCTATCCAGGC -3’  5’-CTCCTTAATGTCACGCACGAT -3’ | IDT integrated DNA technologies |

- 1. **Biological samples**

| **Description** | **Source** | **Identifier** |
| --- | --- | --- |
|  |  |  |

- 1. **Deposited data**

| **Name of repository** | **Identifier** | **Link** |
| --- | --- | --- |
| **Gene Expression Omnibus (GEO)** | **GSE250004** |  |

- 1. **Software**

| **Software name** | **Manufacturer** | **Version** |
| --- | --- | --- |
| **Image J** | **imageJ** | **1.53c** |
| **Prism** | **Graphpad** | **9.4.1** |

- 1. **Other (*e.g*. drugs, proteins, vectors etc.)**

|  |  |  |
| --- | --- | --- |
|  |  |  |

- 1. **Please provide the details of the corresponding methods author for the manuscript:**

| Shuxia Wang, MD,Ph.D., Department of Pharmacology and Nutritional Sciences, University of Kentucky, 900 South Limestone, 583 Wethington Building, Lexington, KY 40536, USA. Phone: 1-859-218-1367; Fax: 1-859-257-3646; Email: [swang7@email.uky.edu](mailto:swang7@email.uky.edu) |
| --- |

**2.0 Please confirm for randomised controlled trials all versions of the clinical protocol are included in the submission. These will be published online as supplementary information.**

| **N/A** |
| --- |
