# Supplementary material for: Platelet-derived thrombospondin 1 promotes immune cell liver infiltration and exacerbates diet-induced steatohepatitis
Source: JHEP Rep. 2024 Jan 26;6(4):101019. doi: 10.1016/j.jhepr.2024.101019 (PMC10918562; doi:10.1016/j.jhepr.2024.101019)

Platelet-derived thrombospondin 1 promotes immune cell liver infiltration and exacerbates diet-induced steatohepatitis

Taesik Gwag,[1,2] Sangderk Lee,[3] Zhenyu Li,[4] Alana Newcomb,[1] Josephine Otuagomah,[1] Steven A. Weinman,[5,6] Ying Liang,[7] Changcheng Zhou,[8] Shuxia Wang[1,2,*]

[1]*Department of Pharmacology and Nutritional Sciences, University of Kentucky, Lexington, KY 40536, United States;* [2]*Lexington Veterans Affairs Medical Center, Lexington, KY 40502, United States;* [3]*Sanders-Brown Center on Aging, University of Kentucky, Lexington, KY 40536, United States;* [4]*Irma Lerma Rangel School of Pharmacy, Texas A&M University, College Station, TX, 77843, United States;* [5]*Department of Internal Medicine, University of Kansas Medical Center, Kansas City, KS 66160, United States;* [6]*Research Service, Kansas City VA Medical Center, Kansas City, MO 64128, United States;* [7]*New York Blood Center, 310 East 72nd nd Street, New York, NY 10065, United States;* [8]*Division of Biomedical Sciences, School of Medicine, University of California, Riverside, CA92521, United States*

JHEP Reports **2024.** https://doi.org/10.1016/j.jhepr.2024.101019

Background & Aims: Recent studies have implicated platelets, particularly α-granules, in the development of non-alcoholic steatohepatitis (NASH). However, the specific mechanisms involved have yet to be determined. Notably, thrombospondin 1 (TSP1) is a major component of the platelet α-granules released during platelet activation. Hence, we aimed to determine the role of platelet-derived TSP1 in NASH.

Methods: Platelet-specific *Tsp1* knockout mice ($TSP1^{\Delta pf4}$) and their wild-type littermates ($TSP1^{F/F}$) were used. NASH was induced by feeding the mice with a diet enriched in fat, sucrose, fructose, and cholesterol (AMLN diet). A human liver NASH organoid model was also employed.

Results: Although TSP1 deletion in platelets did not affect diet-induced steatosis, $TSP1^{\Delta pf4}$ mice exhibited attenuated NASH and liver fibrosis, accompanied by improvements in plasma glucose and lipid homeostasis. Furthermore, $TSP1^{\Delta pf4}$ mice showed reduced intrahepatic platelet accumulation, activation, and chemokine production, correlating with decreased immune cell infiltration into the liver. Consequently, this diminished proinflammatory signaling in the liver, thereby mitigating the progression of NAFLD. Moreover, *in vitro* data revealed that co-culturing TSP1-deficient platelets in a human liver NASH organoid model attenuated hepatic stellate cell activation and NASH progression. Additionally, TSP1-deficient platelets play a role in regulating brown fat endocrine function, specifically affecting Nrg4 (neuregulin 4) production. Crosstalk between brown fat and the liver may also influence the progression of NAFLD.

Conclusions: These data suggest that platelet α-granule-derived TSP1 is a significant contributor to diet-induced NASH and fibrosis, potentially serving as a new therapeutic target for this severe liver disease.

Impact and implications: Recent studies have implicated platelets, specifically α-granules, in the development of non-alcoholic steatohepatitis, yet the precise mechanisms remain unknown. In this study, through the utilization of a tissue-specific knockout mouse model and human 3D liver organoid, we demonstrated that platelet α-granule-derived TSP1 significantly contributes to diet-induced non-alcoholic steatohepatitis and fibrosis. This contribution is, in part, attributed to the regulation of intrahepatic immune cell infiltration and potential crosstalk between fat and the liver. These findings suggest that platelet-derived TSP1 may represent a novel therapeutic target in non-alcoholic fatty liver disease.

© 2024 The Author(s). Published by Elsevier B.V. on behalf of European Association for the Study of the Liver (EASL). This is an open access article under the CC BY license (http://creativecommons.org/licenses/by/4.0/).

Introduction

The pathogenesis of non-alcoholic fatty liver disease (NAFLD)/non-alcoholic steatohepatitis (NASH) is complex.[1] Immune cell-mediated inflammation has emerged as a significant contributor to NAFLD progression and fibrosis development.[2–5] Although macrophages and T cells have been extensively studied in NAFLD, the role of platelets in NASH development has gained recognition.[6–10] In addition to their well-known role in regulating blood hemostasis, accumulating evidence suggests that platelets are significant contributors to NAFLD/NASH.[6–10] Previous studies have reported an association between the platelet count and NAFLD.[11–14] Furthermore, studies using rodent models have demonstrated that anti-platelet therapy can alleviate NASH development[6] and reduce liver damage in patients with NAFLD.[15] A recent study has revealed that platelets accumulate and adhere to Kupffer cells, promoting liver inflammation through a GPIbα-dependent mechanism.[8] Platelet cargo, particularly α-granules, has been implicated in NASH progression.[8] However, the specific constituents of platelet-derived α-granules involved in NASH progression have yet to be fully characterized.

Keywords: Platelet α granule; TSP1; NASH; brown adipocytes; organ crosstalk.
Received 4 October 2023; received in revised form 9 January 2024; accepted 16 January 2024; available online 26 January 2024

* Corresponding author. Address: Department of Pharmacology and Nutritional Sciences, University of Kentucky, Wethington Bldg. Room 583, 900 S. Limestone Street, Lexington, KY 40536, United States; Tel.: +1 859 218 1367, fax: +1 859 257 3646.
E-mail address: swang7@uky.edu (S. Wang).

ELSEVIER
EASL The Home of Hepatology

Thrombospondin 1 (TSP1) is a large glycoprotein with a molecular weight of 420-450 kDa that forms a homotrimeric structure. It constitutes a substantial portion, approximately 20%, of the total content of human platelet α granules, making it one of the most abundant proteins in these granules. TSP1 is rapidly released upon platelet activation[16] and also secreted by various cell types.[17] In addition to regulation of hemostasis, previous studies from our lab established an important role of TSP1 in obesity-associated metabolic diseases including NAFLD/NASH.[18–23] We have demonstrated that TSP1 is an important regulator of monocyte/macrophage functions. It stimulates monocyte/macrophage migration and proinflammatory activation, which contributes to obesity-related chronic inflammation and NAFLD/NASH.[23,24] Although recent advances have illustrated the role of TSP1 in metabolic diseases, the cellular sources of TSP1 that significantly affect the development and progression of NASH remain unresolved. With the recognition of platelets in NASH pathogenesis and the high abundance of TSP1 in platelet α granules, we hypothesize that TSP1 from platelet sources might be a significant contributor to NASH. The aim of this current study was to test this hypothesis.

In this study, for the first time, we investigated the role of platelet-derived TSP1 in a diet-induced NASH mouse model using platelet-specific TSP1-deficient mice as well as human NASH organoids. Although TSP1 deletion in platelets did not protect against diet-induced steatosis, it significantly attenuated liver inflammation and fibrosis, providing strong evidence that platelet-derived TSP1 is an important contributor to NAFLD progression.

Material and methods

Mice and diet

All experiments involving mice were approved by the University of Kentucky Institutional Animal Care and Use Committee. Animals were housed in standard cages at 22 °C under a 12:12-h light-dark cycle. To produce platelet-specific TSP1-deficient mice ($TSP1^{\Delta pf4}$), *Tsp1* floxed mice generated in our laboratory[24] ($TSP1^{F/F}$) were crossbred with *Pf4-Cre* mice from Jax Lab for two generations. Male $TSP1^{\Delta pf4}$ mice and littermate controls ($TSP1^{F/F}$) were used in our studies. For dietary models, mice were fed a low-fat diet (LF, 10% kcal, D1250B, Research Diets, Inc, NJ) or AMLN diet (40% fat; 22% fructose and 2% cholesterol, D09100301, Research Diets, Inc, NJ) for 16 weeks (to induce simple steatosis) and 32 weeks (to induce NASH).

Metabolic analysis

Body weight was monitored weekly. Body composition (*e.g.*, fat mass and lean mass) was measured using Echo-MRI. Glucose tolerance test and data analysis were performed as previously described.[25] Indirect calorimetry was performed by placing mice in the Sable Promethion Metabolic Chamber.

Liver histology analysis and immunohistochemical staining

Liver histology (H&E staining) and the NAFLD activity score were assessed as previously described.[26–28] Sirius Red or Trichrome staining, provided by a Pathology Core at the University of Kentucky, was used to analyze liver fibrosis. For liver immunohistochemical staining, liver paraffin sections were stained with anti-CD41 antibody (Thermo Fisher Scientific; MA, USA), anti-neutrophil antibody [7/4] (ab53457) (Abcam; Cambridge CB20AX, UK), anti-p-selectin antibody (Novus Biologicals, CO, USA), anti-CD4 antibody (Abcam; Cambridge CB20AX, UK), or anti-CD8 antibody (Novus Biologicals, CO, USA), followed by incubation with biotinylated secondary antibody, peroxidase substrate diaminobenzidine (Vector Lab) and counterstaining with hematoxylin. Images were captured by a Nikon Eclipse 55i microscope. Semi-quantification of neutrophil, CD4, or CD8 positive cells in liver sections was performed as previously described.[29]

Lipid analysis

Total lipids from frozen liver were extracted as previously described.[30] Liver and plasma triacylglycerol and total cholesterol levels were measured enzymatically using kits from Wako Chemicals (Richmond, USA). Additionally, plasma from multiple mice (n = 5) in each group was pooled, and fast-performance liquid chromatography was used to determine plasma lipoprotein distribution.[31]

Blood parameter analysis

At the end of the study, blood hematology analysis was performed using the Hemavet 950FS. Plasma alanine aminotransferase (ALT) levels were measured using ALT assay kits (Sigma-Aldrich, MD, USA). Plasma insulin levels were measured using an ELISA kit (Crystal Chem, IL, USA). PF4 levels in platelet-rich plasma or platelet-poor plasma were measured using a mouse CXCL4/PF4 immunoassay kit (R&D system; MN, USA).

Liver RNA-Seq transcriptome sequencing

Liver bulk RNA sequencing was performed by Novogene (Sacremento, CA) NovaSeq PE150, for all 16 liver samples (n = 4 mice/group), with an average read depth of 6G reads per sample. Reads were aligned using STAR 2.7.8a and quantified using the mouse reference genome mm10. Analysis of differentially expressed genes (DEGs) between groups of interest was conducted using the DESeq2 algorithm. DEGs were selected if the adjusted p value was less than 0.05 and the absolute value of log-fold change was higher than 0.25. Based on the identified DEGs between groups of interest, enrichment analyses of gene ontology terms, the KEGG pathway, and the Reactome pathway were performed using Cluster Profiler R program package. Enrichment analysis results were filtered out if the adjusted p value was greater than 0.05. The data have been deposited in the Gene Expression Omnibus with accession number GSE250004.

Real-time quantitative PCR

Total RNA from the liver and other tissues was extracted, reverse transcribed to cDNA, subjected to quantitative PCR analysis using a MyiQ Real-time PCR Thermal Cycler (Bio-Rad) with SYBR Green PCR Master Kit (Qiagen, Valencia, CA) and normalized to β-actin mRNA levels as previously described.[23] All the primer sequences utilized in this study are listed in Table S1.

Western blotting

Proteins obtained from the liver or other tissues were separated using SDS-PAGE and subsequently transferred onto a nitrocellulose membrane. To determine protein expression, immunoblotting was performed using the following antibodies: anti-TSP1 (Novus Biologicals), anti-α-SMA (smooth muscle actin) (Sigma-Aldrich), or anti-β-actin (Sigma-Aldrich). The membranes were incubated with primary antibodies and then with appropriate secondary antibodies conjugated with horseradish

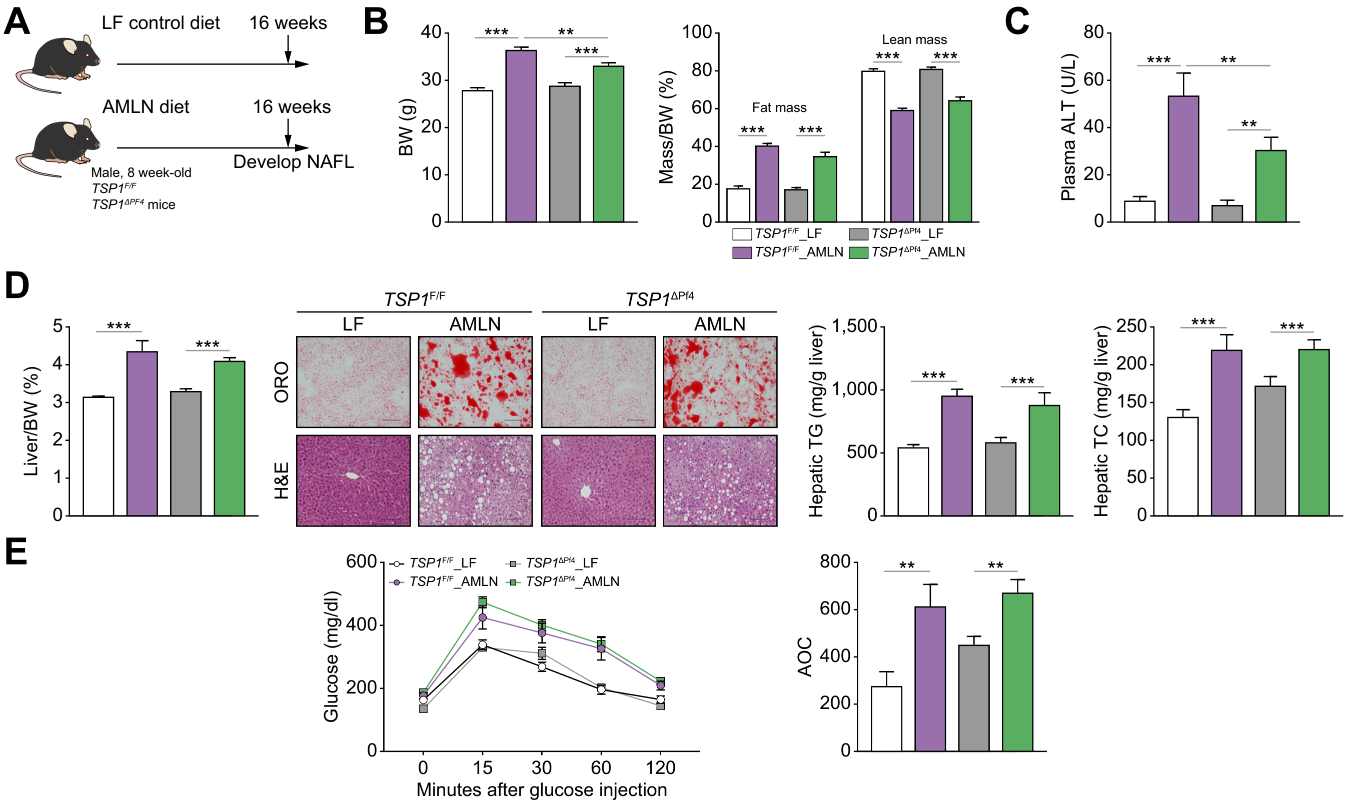

Fig. 1. Deletion of TSP1 in platelets did not protect mice from the 16-week AMLN diet-induced hepatic steatosis. (A) Schematic diagram of experimental setting for 16 weeks LF or AMLN diet feeding; (B) Body weight and body composition were measured at 16 weeks after LF or AMLN diet feeding; (C) Plasma ALT levels; (D) Liver weight/BW ratio, representative ORO (top panel) and H&E (bottom panel) staining of liver sections from each group (Scale bar = 100 μm), and hepatic TG and TC levels; (E) Glucose tolerance test and AOC were analyzed. Data are represented as mean ± SE (n=6-10 mice/group); one-way ANOVA with Tukey's *post hoc* test. $^{**}p$ <0.01 and *** p <0.001. ALT, alanine aminotransferase; AOC, area of the curve; BW, body weight; HOMA-IR, homeostatic model assessment of insulin resistance; LF, low fat; ORO, Oil red O; TC, total cholesterol; TG, triglycerides.

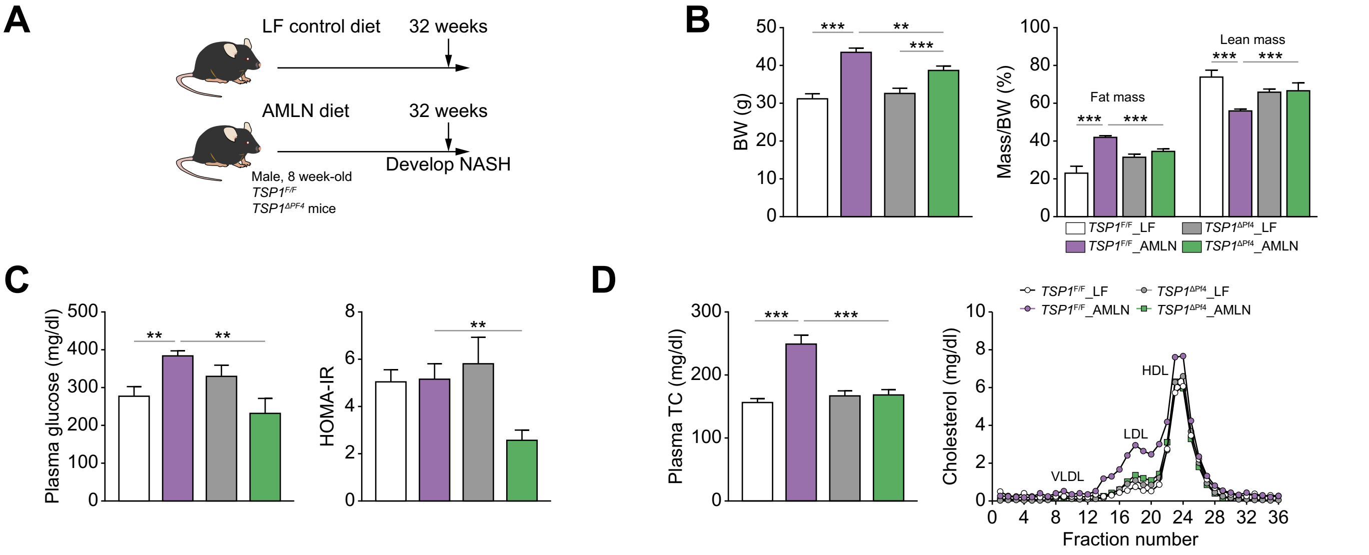

Fig. 2. Deletion of TSP1 in platelets attenuated diet-induced hyperlipidemia, and improved glucose homeostasis in mice after 32-week AMLN diet feeding. (A) Schematic diagram of experimental setting for 32 weeks LF or AMLN diet feeding; (B) Body weight and body composition were measured at 32 weeks after LF or AMLN diet feeding; (C) Plasma glucose and HOMA-IR in postprandial condition; (D) Total plasma cholesterol levels and the plasma lipoprotein contribution by fast-performance liquid chromatography. Data are represented as mean ± SE (n = 4-7 mice/group); one-way ANOVA with Tukey's *post hoc* test. $^{*}p$ <0.05, $^{**}p$ <0.01 and $^{***}p$ <0.001. BW, body weight; HOMA-IR, homeostatic model assessment of insulin resistance; LF, low fat; NASH, non-alcoholic steatohepatitis; TC, total cholesterol.

peroxidase. Labelled proteins were visualized using an enhanced chemiluminescence system (Pierce).

3D human NASH organoid model with or without platelet co-culture

A 3D human NASH *in vitro* model was previously established in our lab.[29] Briefly, human hepatocytes (ATCC), THP1-derived macrophages (ATCC) and human stellate cells (Zenbio) were co-cultured to form 3D spheroids and then treated with NASH-inducing media (DMEM media containing 1% BSA, palmitate [0.5 mM], high glucose [30 mM] and lipopolysaccharide [2 μg/ml]) for 5 days to induce the proinflammatory and profibrogenic phenotype. To determine the effect of TSP1-depleted platelets on NASH progression in liver organoids, purified platelets ($1x10^4$) from wild-type (WT) or TSP1-deficient mice were added into 3D spheroids in the presence of NASH-inducing media for 5 days. After treatment, cells were harvested, and the expression of genes related to inflammation and fibrosis was determined by real-time PCR. Additionally, organoids were harvested for immunofluorescence staining with anti-α-SMA (Sigma, 1:100) and then secondary antibody-mouse-Alexa488. After staining, slides were mounted with mounting medium containing DAPI. The images were captured with the Nikon A1R confocal microscope.

Statistical analysis

Statistical analysis was performed using Prism version 9.0 (GraphPad Software, San Diego, CA, USA). Data are expressed as mean values ± SE. Two-tailed Student's *t* test was used to determine statistical significance between the two groups. One-way ANOVA followed by Tukey's multiple comparison test or two-way ANOVA followed by Tukey's multiple comparison test was used for multi-group comparisons.

Results

Platelet-specific TSP1 deficiency failed to protect mice against diet-induced steatosis

TSP1 deficiency in platelets of *TSP1*$^{\Delta Pf4}$ mice was confirmed through PCR and western blotting (Fig. S1A-C). TSP1 deficiency did not affect blood platelet counts (Fig. S1D, Table S2). Platelet aggregation and mouse tail bleeding time were also measured as

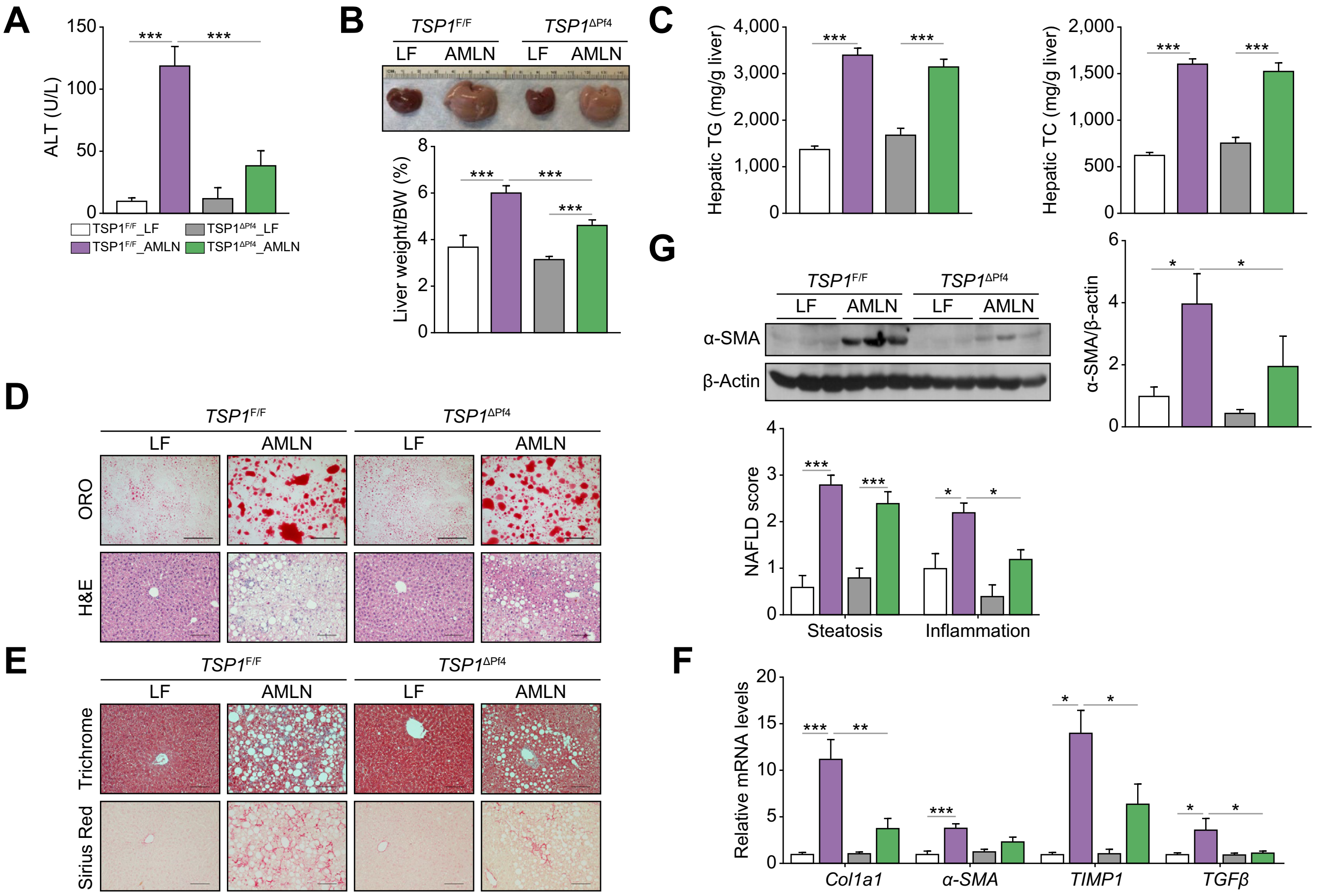

Fig. 3. Deletion of TSP1 in platelets attenuated NASH and liver fibrosis induced by 32-week AMLN diet feeding induced. (A) Plasma ALT levels; (B) Representative liver image and weight from four groups of mice; (C) Hepatic TG and TC levels; (D) Representative ORO (top panel) and H&E (bottom panel) staining of liver sections from four groups of mice (Scale bar = 100 μm). The NAFLD scores were determined from H&E staining; (E) Representative image of Trichrome staining (top panel) and Sirius red staining (bottom panel) from four groups of mice (Scale bar = 100 μm). Hepatic fibrosis gene expressions were determined by qPCR (F) and α-SMA protein expression levels were determined by immunoblotting (G). Data are represented as mean ± SE (n = 5 mice/group); one-way ANOVA (for C) and G) and two-way ANOVA (for D and F) with Tukey's multiple comparisons test. **p* <0.05, ***p* <0.01, and *** *p* <0.001. ALT, alanine aminotransferase; BW, body weight; LF, low fat; ORO, Oil red O; TC, total cholesterol; TG, triglycerides.

previously described[32] and were comparable between WT and knockout (KO) mice (Fig. S1E,F), Together, these data suggest that deletion of TSP1 in platelets does not affect hemostasis under physiological conditions.

To determine whether platelet TSP1 deficiency affects NAFLD development and progression, a mouse model of AMLN diet-induced NASH (obesity and insulin resistance)[33] was utilized. Simple steatosis was induced by feeding mice with the AMLN diet for 16 weeks. We observed that AMLN-fed $TSP1^{\Delta Pf4}$ mice had slightly reduced body weight compared to AMLN-fed $TSP1^{F/F}$ mice (Fig. 1A,B). Plasma ALT levels were also reduced in the AMLN-fed $TSP1^{\Delta Pf4}$ mice (Fig. 1C). However, liver weight/body weight ratio, liver fat accumulation (demonstrated by Oil Red O staining), hepatic total cholesterol and triglyceride measurements, and glucose tolerance tests were comparable between the two genotypes after AMLN diet feeding (Fig. 1D,E). These data suggest that platelet TSP1 deficiency did not affect the development of diet-induced steatosis.

Platelet-specific TSP1 deficiency protected mice against diet-induced steatohepatitis

To determine whether platelet TSP1 deficiency affects the progression of simple steatosis to NASH, we conducted additional experiments over 32 weeks. We observed that AMLN-fed $TSP1^{\Delta Pf4}$ mice exhibited reduced body weight and fat mass compared to control $TSP1^{F/F}$ mice (Fig. 2A,B). However, food intake and energy expenditure were similar between the two genotypes (data not shown). Interestingly, white fat tissue mass, including epididymal fat tissue (eWAT) and subcutaneous fat tissue (sWAT), was reduced in the AMLN-fed $TSP1^{\Delta Pf4}$ mice (Figs S2A and S3A). Adipocyte size and distribution analysis showed that adipocytes from eWAT and sWAT of AMLN-fed $TSP1^{\Delta Pf4}$ mice tended to be smaller than those of control $TSP1^{F/F}$ mice (Figs S2B and S3B). In addition, eWAT and sWAT from AMLN-fed $TSP1^{\Delta Pf4}$ mice exhibited increased expression of lipolysis-related genes (*e.g.* ATGL, LPL, and HSL) (Figs S2C and S3C). Platelet infiltration into eWAT and sWAT was comparable between control $TSP1^{F/F}$ and $TSP1^{\Delta Pf4}$ mice, as shown by immunofluorescence staining (Figs S2D and S3D). Moreover, an *in vitro* study involving the co-culturing of white preadipocytes (3T3-L1) with platelets isolated from WT or *Tsp1* KO mice demonstrated that platelet-derived TSP1 did not significantly affect white pre-adipocyte proliferation (Fig. S4A). Interestingly, the co-culturing of *Tsp1* KO platelets with 3T3-L1 cells seemed to stimulate their differentiation into adipocytes, as demonstrated by increased Oil Red O-positive staining and elevated expression of marker genes for differentiation, such as PPAR-r, AP2, *etc.* (Fig. S4B,C). These two findings, increased adipogenesis and an upregulated lipolysis pathway, appear paradoxical in the regulation of fat mass. However, the observed reduction in white fat mass in KO mice ($TSP1^{\Delta Pf4}$) may depend on the relative rates of these opposing forces. In addition, the reduced expansion (hypertrophy) of adipocytes in KO mice may also contribute to the observed decrease in fat mass. This warrants further investigation in the future.

We also investigated the alteration of brown fat tissue (BAT) in two genotypes. We found that AMLN-fed $TSP1^{\Delta Pf4}$ mice exhibited reduced BAT mass and whitening, as well as smaller size of brown adipocytes (Fig. S5A,B). BAT from AMLN-fed $TSP1^{\Delta Pf4}$ mice showed increased expression of UCP1 and Nrg4 (neuregulin 4) (Fig. S5C), accompanied by reduced platelet accumulation in BAT (Fig. S5D). Nrg4 is a secreted protein enriched in BAT and has been shown to be involved in NAFLD/NASH progression.[34,35] To further determine the effect of platelet-derived TSP1 on Nrg4 expression in brown adipocytes, a co-culture study was performed using a brown adipocyte cell line (T37i) and platelets. As shown in Fig. S5E, co-culturing of brown adipocytes with *Tsp1* KO platelets resulted in increased Nrg4 expression and secretion compared to WT platelets. There was a trend towards an increase in plasma Nrg4 levels in $TSP1^{\Delta Pf4}$ mice compared to control $TSP1^{F/F}$ mice (Fig. S5F). These data suggest a role of TSP1-deficient platelets in regulating the endocrine function of BAT. Through secreted Nrg4 protein, BAT may communicate with the liver in AMLN-fed $TSP1^{\Delta Pf4}$ mice, impacting NAFLD progression. Collectively, these data reveal a previously unrecognized role of platelet TSP1 in regulating white fat lipid metabolism and brown fat endocrine function. Whether these effects have a potential impact on NASH development in the current model remains to be determined.

Additionally, AMLN-fed $TSP1^{\Delta Pf4}$ mice showed improved glucose homeostasis and lower plasma total cholesterol and LDL cholesterol (Fig. 2C,D). Furthermore, in analyzing the blood profile, we observed an upregulation of white blood cells, particularly lymphocyte and monocyte populations, in AMLN-fed $TSP1^{F/F}$ mice, whereas a decrease in these cell counts was observed in AMLN-fed $TSP1^{\Delta Pf4}$ mice (Table S3).

Furthermore, we analyzed liver phenotypes. As shown in Fig. 3A, AMLN-fed $TSP1^{\Delta Pf4}$ mice exhibited less liver injury, as indicated by the reduced ALT levels. Liver weight/body weight ratio was reduced in AMLN-fed $TSP1^{\Delta Pf4}$ mice, while liver triglycerides and total cholesterol were comparable between the two genotypes (Fig. 3B,C). Histological analysis revealed decreased NAFLD scores, particularly inflammation, in AMLN-fed $TSP1^{\Delta Pf4}$ mice (Fig. 3D). The reduction in liver fibrosis in AMLN-fed $TSP1^{\Delta Pf4}$ mice was confirmed by Masson's trichrome and

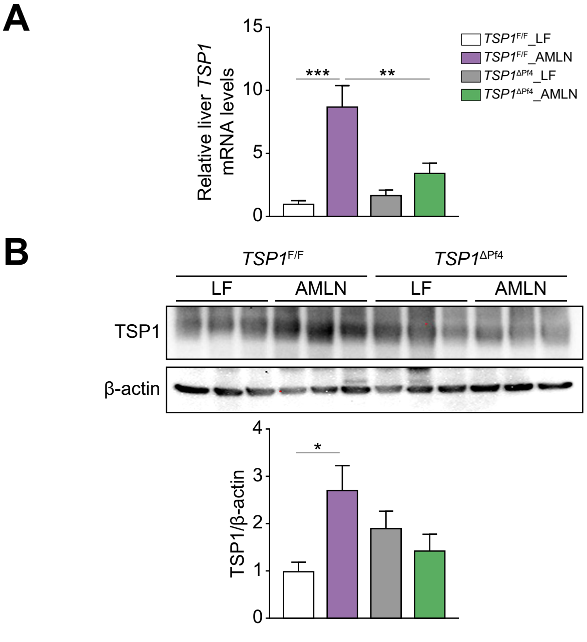

Fig. 4. Deletion of TSP1 in platelets attenuated liver TSP1 levels in 32-week AMLN diet-fed mice. (A) Expression of TSP1 in livers from four groups of mice by qPCR and (B) liver TSP1 protein levels by immunoblotting. Data are represented as mean ± SE (n = 5-6 mice/group); one-way ANOVA with Tukey's *post hoc* test. $^*p$ <0.05, $^{**}p$ <0.01, and *** p <0.001. LF, low fat.

Sirius Red staining, as well as qPCR (Fig. 3E,F). Reduced hepatic stellate cell activity was revealed by decreased levels of liver α-SMA protein (Fig. 3G). Taken together, these findings demonstrate that while platelet-derived TSP1 does not appear to influence the development of steatosis, it plays a significant role in the progression from steatosis to NASH and liver fibrosis.

Intrahepatic platelet accumulation, chemokine production and immune cell infiltration were attenuated in AMLN-fed $TSP1^{\Delta Pf4}$ mice

The potential mechanisms underlying the protective effect of platelet-specific TSP1 deficiency on diet-induced NASH and liver fibrosis were investigated (Fig. 3). We found that liver *Tsp1* mRNA levels were significantly reduced, and TSP1 protein levels trended towards a reduction in AMLN-fed $TSP1^{\Delta Pf4}$ mice (Fig. 4). Furthermore, we conducted whole-liver transcriptome analysis. In control $TSP1^{F/F}$ mice, a comparison of the AMLN and LF diets revealed a significant upregulation of 1,726 genes and a significant downregulation of 832 genes. When comparing AMLN-fed $TSP1^{F/F}$ to the AMLN-fed $TSP1^{\Delta Pf4}$ mice, there were 1,293 upregulated genes and 2,226 downregulated genes. We performed an enriched pathway analysis. As shown in Fig. 5, KEGG-enriched pathway analysis revealed several specific downregulated pathways in $TSP1^{\Delta Pf4}$ mice, including cytokine-cytokine receptor interaction and chemokine signaling pathways, among others. Consistently, gene ontology pathway analysis identified downregulated pathways related to negative regulation of the immune system process, tumor necrosis factor superfamily cytokine production, leukocyte cell-cell adhesion, leukocyte chemotaxis, cytokine or chemokine activity, and more. These findings are in line with the NAFLD score data (Fig. 3D) and confirm the reduced liver inflammation observed in the AMLN-fed $TSP1^{\Delta Pf4}$ mice. This further suggests that platelet *Tsp1* deletion suppresses immune cell trafficking in the liver. As expected, the expression of chemokines such as CCL2, CXCL1, CXCL2, CXCL4 (PF4), and CXCL5 in the liver was reduced in AMLN-fed $TSP1^{\Delta Pf4}$ mice compared to that in AMLN-fed $TSP1^{F/F}$ mice (Fig. 6A,B). This reduction was accompanied by decreased liver accumulation of immune cells such as neutrophils, CD4, and CD8 T cells in AMLN-fed $TSP1^{\Delta Pf4}$ mice (Fig. 6C,D).

We also conducted Reactome analysis of the liver RNA-seq data and identified that genes associated with platelet

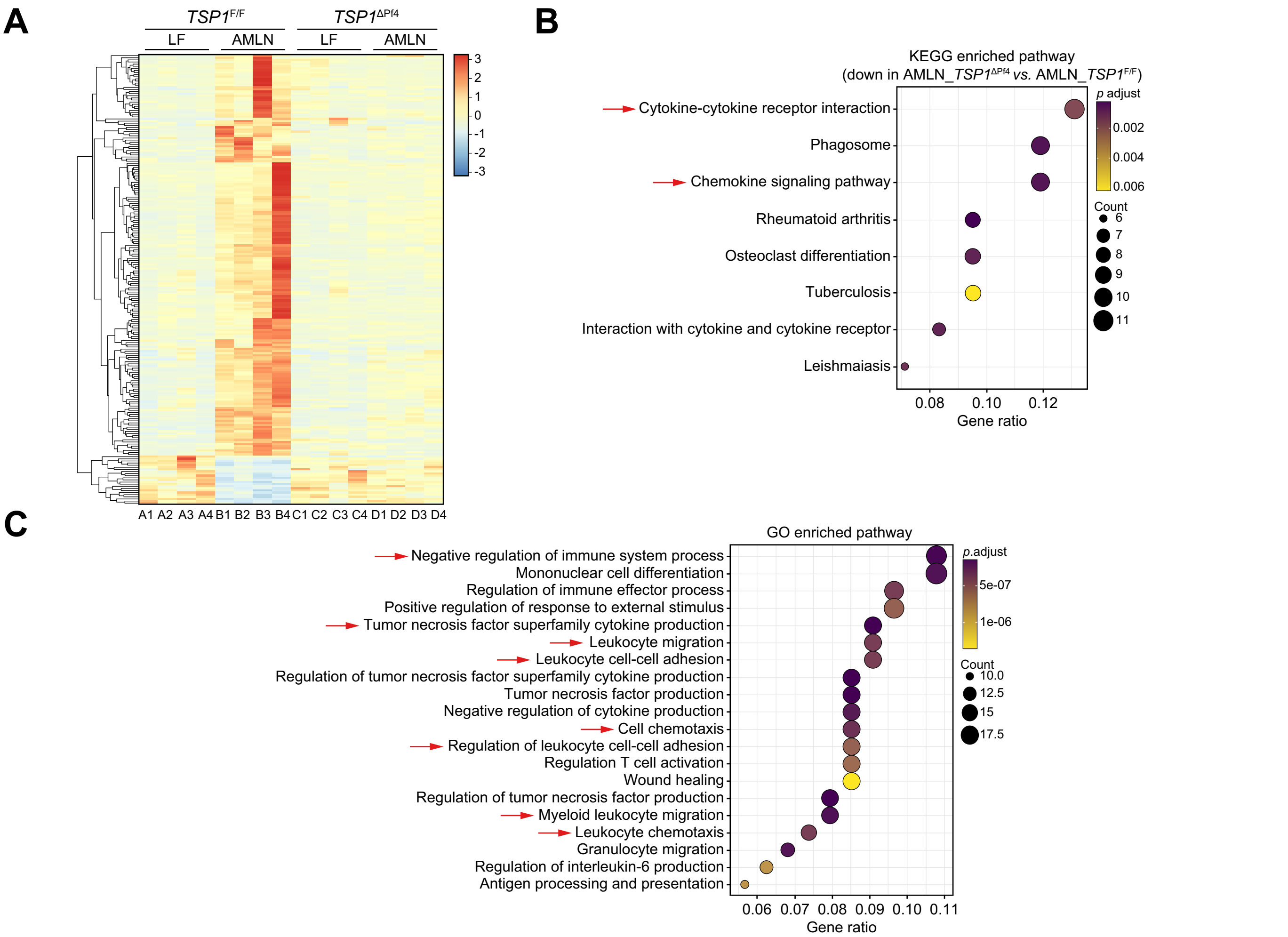

Fig. 5. Liver RNA-seq analysis from 32-week AMLN diet-fed mice. (A) Heatmap of liver transcriptome analysis from four groups of mice (n = 4 mice/group). The color spectrum from blue to red represents the gene expression intensity from low to high, respectively. Based on hierarchical clustering of genes that were significantly upregulated in AMLN_$TSP1^{F/F}$ compared to LF_$TSP1^{F/F}$ and downregulated in AMLN_$TSP1^{\Delta Pf4}$ compared to AMLN_$TSP1^{F/F}$, KEGG pathway (B) and GO pathway (C) were analyzed. GO, gene ontology; LF, low fat.

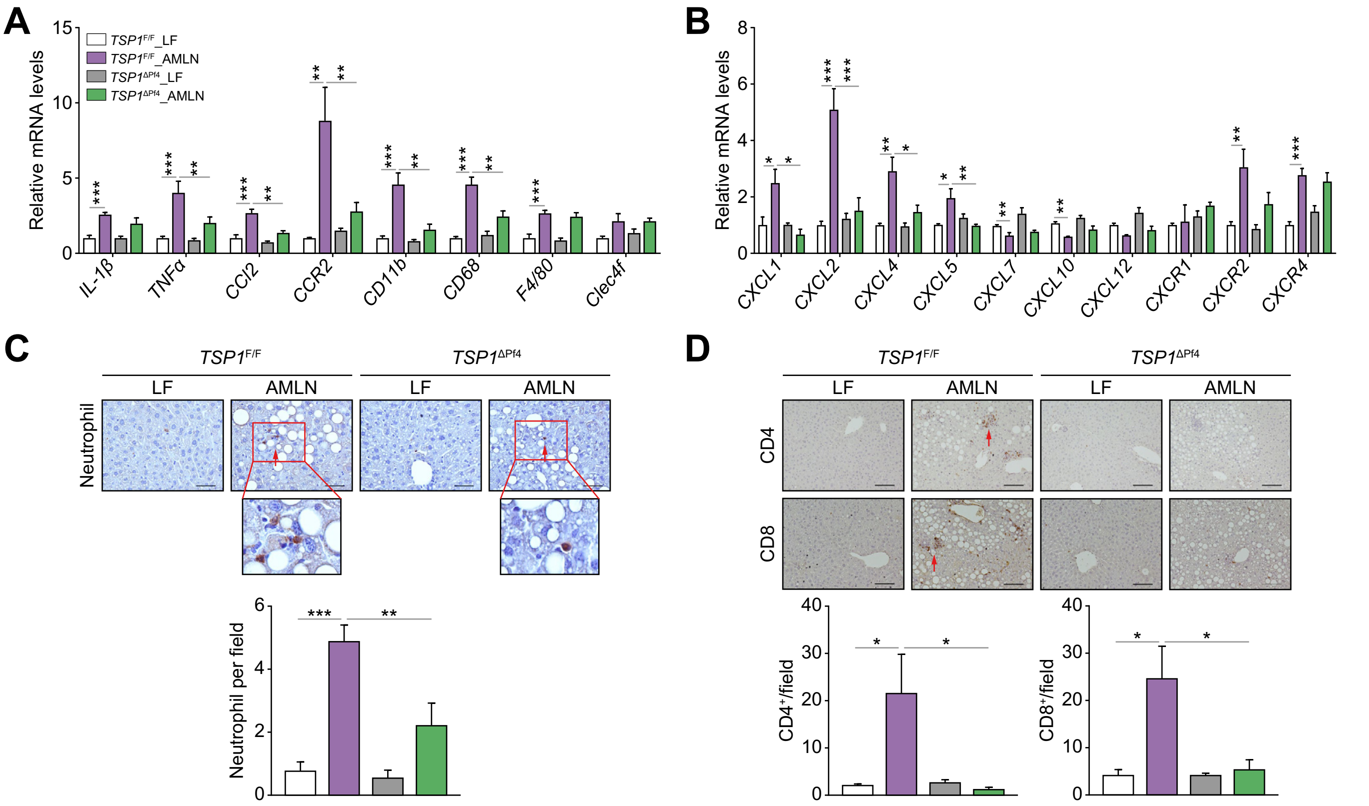

Fig. 6. Deletion of TSP1 in platelets attenuated liver immune cell infiltration and proinflammatory cytokine production in 32-week AMLN diet-fed mice. (A) Expression of genes related to inflammation and macrophages markers in livers from four groups of mice by qPCR; (B) Expression of genes related to chemokines and receptors in livers by qPCR; (C) Representative images of neutrophil immunohistochemistry staining in liver sections and semi-quantification data; (D) Representative image of CD4 (top panel) and CD8 (bottom panel) immunohistochemistry staining in liver sections and semi-quantification data; scale bar = 100 μm. Data are represented as mean ± SE (n = 5-6 mice/group); one-way ANOVA (for C and D) and two-way ANOVA (for A and B) with Tukey's multiple comparisons test. **p* <0.05, ***p* <0.01, ****p* <0.001. LF, low fat.

activation, signaling, or aggregation (*e.g.*, orm2, CD63, PF-4, plek, pik3r5) were upregulated in AMLN-fed $TSP1^{F/F}$ livers but downregulated in AMLN-fed $TSP1^{\Delta Pf4}$ livers (Fig. 7A,B). Additionally, we confirmed that platelet-rich or platelet-poor plasma isolated from AMLN-fed $TSP1^{\Delta Pf4}$ mice exhibited reduced production of PF-4 (a chemotactic factor for neutrophils and monocytes[36]) compared to AMLN-fed $TSP1^{F/F}$ mice (Fig. 7C). Moreover, the results of liver immunohistochemical staining demonstrated a reduction in intrahepatic platelet accumulation and activation, as evidenced by decreased co-staining of CD41 and p-selectin (a marker for activated platelets) in AMLN-fed $TSP1^{\Delta Pf4}$ mice (Fig. 7D,F). Reduced interaction between intrahepatic platelets and macrophages was also observed in AMLN-fed $TSP1^{\Delta Pf4}$ mice (Fig. 7E). Collectively, these findings suggest that TSP1-depleted platelets are less active and secrete fewer chemokines, which may contribute to reduced infiltration of immune cells in the liver.

TSP1-deficient platelets inhibited the development of inflammation and fibrosis in 3D human NASH organoids

To further validate the inhibitory effect of TSP1-deficient platelets on the development of NASH and demonstrate its relevance in a human context, we utilized a 3D human NASH *in vitro* model that was previously established in our studies.[29] In this model, human hepatocytes, macrophages, and stellate cells are co-cultured to form scaffold-free 3D spheroids. WT or TSP1-deficient platelets were added to 3D spheroids, which were then treated with NASH-inducing media for 3-5 days. After the treatment, cells were harvested for qPCR analysis. As shown in Fig. 8A, the NASH media stimulated the expression of proinflammatory and profibrotic genes, such as IL-1β, α-SMA, and TIMP1. However, the addition of TSP1-deficent platelets attenuated the upregulation of these genes, indicating a suppression of inflammation and fibrosis. Moreover, confocal fluorescence images demonstrated a reduction in α-SMA-positive staining in the 3D spheroids with TSP1-deficient platelets (Fig. 8B). These results suggest that TSP1-deficient platelets have the potential to attenuate inflammation and fibrosis in NASH.

Discussion

In this study, we investigated the role of platelet-α granule-derived TSP1 in the development and progression of NAFLD. To achieve this, we used both an AMLN diet-induced NASH mouse model and a 3D human NASH organoid model. Our findings demonstrate that deletion of TSP1 in platelets does not provide protection against obesity-associated steatosis in mice. However, it confers protection against obesity-associated NASH and liver fibrosis. Mechanistically, platelets lacking TSP1 exhibited reduced intrahepatic accumulation, activation, and chemokine secretion, resulting in decreased infiltration of immune cells into the liver and subsequently reduced stellate

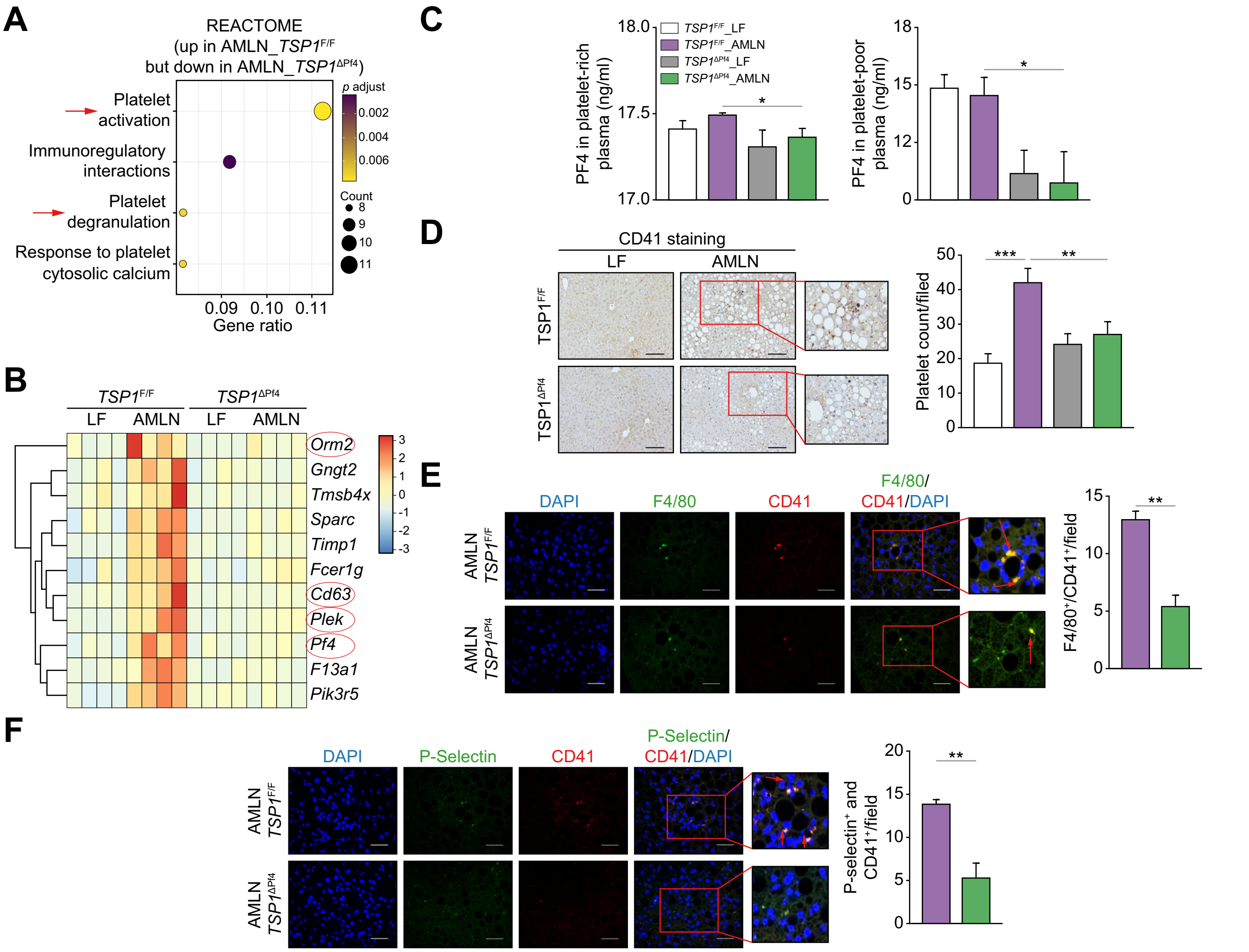

Fig. 7. Deletion of TSP1 in platelets reduced intrahepatic platelet accumulation and activation in 32-week AMLN diet-fed mice. (A) Reactome pathway analysis of liver transcriptome data from Fig. 5A; (B) Hierarchical clustering analysis of genes in platelet activation, signaling and aggregation category from Reactome pathway. The color spectrum from blue to red represents the gene expression intensity from low to high, respectively; (C) CXCL4/PF4 levels from both platelet-rich plasma and platelet-poor plasma was measured; (D) Representative image of CD41 immunohistochemistry staining in liver sections (scale bar = 100 µm) and semi-quantification data; (E) Representative immunofluorescence image of F4/80 and CD41 co-staining in liver sections (scale bar = 100 µm). Nuclei were stained with DAPI; (F) Representative immunofluorescence image of P-Selectin and CD41 co-staining in liver sections and semi-quantification data, Scale bar = 100 µm. Nuclei were stained with DAPI. Data are represented as mean ± SE (n = 5 mice/group); one-way ANOVA with Tukey's *post hoc* test. *p <0.05, **p <0.01, ***p <0.0001. LF, low fat.

cell activation. Collectively, these findings indicate that platelet-derived TSP1 plays a crucial role in the NAFLD progression and may serve as a potential therapeutic target for this disease.

TSP1 represents approximately 20% of the total protein in platelet α-granules, from where it is released upon platelet activation. As one of the most abundant α-granule proteins, platelet TSP1 has been shown to play a role in the regulation of thrombosis and hemostasis. Previous research has demonstrated that platelet-released TSP1 regulates platelet adhesion and collagen-dependent thrombosis stabilization through its interaction with its receptor, CD36, *in vitro*.[37] TSP1 has also been shown to stimulate platelet aggregation by inhibiting the anti-thrombotic activity of nitric oxide/cGMP signaling[38] or contribute to von Willebrand factor-dependent thrombus formation.[39] Additionally, recent research suggests that platelet-derived TSP1 plays a role in promoting hemostasis and regulating thrombosis *in vivo* by modulating platelet cAMP signaling at sites of vascular injury.[40] However, it is important to note that these studies utilized global TSP1-deficient mice, which limits their ability to definitively determine the specific role of platelet-derived TSP1 in hemostasis. In this study, we have generated

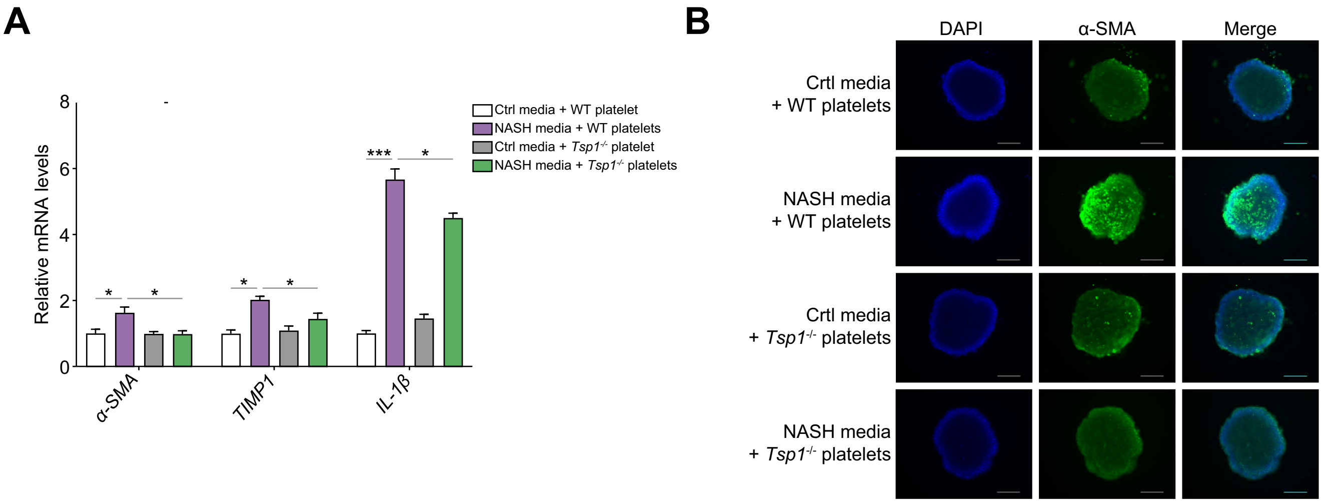

Fig. 8. Deletion of TSP1 in platelets attenuated inflammation and stellate cell activation in a human NASH organoid model. *In vitro* 3D human NASH model was established by co-culturing human hepatocyte/THP-1-derived macrophages/human stellate cells to form spheroids. Spheroids were treated with control media or NASH-inducing media for 5 days and then platelets were added from WT or *Tsp1*$^{-/-}$ mice for an additional 5 days. (A) Gene expression was determined by qPCR; B) Representative immunofluorescent images from a spheroid (blue = DAPI; green = αSMA). Scale bar = 200 μm; Data are represented as mean ± SE (n = 3 separate experiments); two-way ANOVA with Tukey's multiple comparisons test. **p* <0.05; ****p* <0.001. NASH, non-alcoholic steatohepatitis; WT, wild-type.

platelet-specific *Tsp1* KO mice by crossbreeding TSP1-floxed mice with *pf4-cre* mice. The *pf4-cre* mouse model is a commonly used Cre driver for generating megakaryocyte/platelet-specific KO mice, although a more stringent megakaryocyte lineage-specific expression model, Gp1ba-Cre mice, has recently been developed by Nagy *et al.*[41] In our characterization of $TSP1^{\Delta pf4}$ (8-week-old male mice), we did not observe any obvious abnormalities. Interestingly, $TSP1^{\Delta pf4}$ mice exhibited normal blood platelet counts, platelet aggregation, and tail bleeding times compared to WT control mice. These findings suggest that platelet-derived TSP1 is not a significant contributor to hemostasis under physiological conditions, but further investigation is warranted to fully elucidate its role in hemostasis and thrombosis.

Our current study provides compelling evidence supporting the involvement of platelet-derived TSP1 in the regulation of diet-induced NASH development and progression. Previous studies from our lab and others have established the role of TSP1 in NAFLD/NASH.[19,20,23,42,43] This study takes a step further by advancing our understanding of the specific contribution of TSP1 derived from platelets to NAFLD progression. We found that platelet-derived TSP1 does not influence the development of diet-induced steatosis. In contrast, our findings suggest that in the late stages of NAFLD, activated platelets within the steatotic liver release TSP1 and other chemokines (*e.g.* CXCL1, CXCL2, and CXCL4) to attract immune cells (*e.g.* monocytes, neutrophils, and T cells) to infiltrate into the liver.[18,36,44] Recruitment of immune cells further amplifies proinflammatory signaling within the liver, leading to stellate cell activation and the subsequent development of liver fibrosis. Consistent with the *in vivo* study, a 3D human NASH *in vitro* model further demonstrated that TSP1-deficient platelets have the potential to attenuate inflammation and fibrosis directly or indirectly through secreted factors to affect liver cell functions. These findings open new avenues for potential platelet-based therapy in NASH, particularly considering the minimal impact of TSP1-deficient platelets on hemostasis.

It is known that anucleate platelets cannot be directly modified using traditional genetic approaches. Instead, hematopoietic stem cell-derived megakaryocytes, the nucleated precursor to platelets, can be genetically manipulated.[45] Hematopoietic stem cells can be isolated from bone marrow, peripheral blood, and cord blood after birth. Furthermore, recent studies have demonstrated the efficient differentiation of blood CD34+ hematopoietic progenitors into megakaryocytes.[46] Additionally, CRISPR/Cas9 transfection can perform efficient gene editing in CD34+ cells.[47] These exciting recent advances in human platelet functional studies are truly promising, making it possible for us to target TSP1 in platelets for therapeutic purposes. This could be achieved by isolating CD34+ hematopoietic stem cells from patient blood, performing *in vitro* CRISPR-mediated *TSP1* deletion, differentiating these cells into mature megakaryocytes, and producing TSP1-depleted platelets. Subsequently, these gene-modified platelets could be transfused back into the same patient for therapeutic purposes.

Additionally, our current study has unveiled a previously unknown role of platelet-derived TSP1 in regulating brown fat endocrine/paracrine function. Brown fat has been recognized as a source of various molecules known as batokines, including IL-6, FGF21, and Nrg4, which possess the ability to communicate with other organs, influencing systemic metabolism.[48] Among the batokines analyzed in our study, we found that Nrg4 expression was notably upregulated in brown fat from $TSP1^{\Delta pf4}$ mice. *In vitro* studies further demonstrated a negative regulatory effect of platelet-derived TSP1 on Nrg4 production in brown adipocytes. Nrg4 is known to establish a link between brown fat and the liver. Interestingly, previous research has demonstrated the crucial role of hepatic Nrg4 signaling in the

progression from steatosis to NASH.[34] This finding raises the intriguing possibility that the protective effect of TSP1-deficient platelets on NASH progression could also be partially mediated by the crosstalk between brown fat and the liver. However, this relationship warrants further investigation in future studies. Our discovery of this potential novel interaction between platelet-derived TSP1, Nrg4, brown fat, and the liver may add a new layer of complexity to our understanding of NASH progression. Unraveling the interplay between these factors may lead to new therapeutic approaches for NASH and related metabolic disorders.

In summary, our study utilizing tissue-specific *Tsp1* KO mice offers compelling evidence linking platelet-derived TSP1 to the pathogenesis of NAFLD/NASH progression. This novel finding sheds light on the intricate interplay between platelet function, obesity, and liver disease. Importantly, our findings suggest that platelet-derived TSP1 holds promise as a potential therapeutic target for managing NAFLD/NASH.

Abreviations

NAFLD, nono-alcoholic fatty liver disease; NASH, non-alcoholic steatohepatitits; Nrg4, neuregulin 4; PF4, platelet factor 4; TSP1, thrombospobdin 1; $TSP1^{F/F}$, TSP1 floxed mice; $TSP1^{\Delta Pf4}$, mice with TSP1 depleted in platelets.

Financial support

This work was supported by the Department of Veterans Affairs Merit Review Award (BX004252 to SW), the National Institutes of Health (NIH) Grant (DK131786, to SW), and an Institutional Development Award (IDeA) from the National Institute of General Medical Sciences under grant number P30 GM127211.

Conflict of interest

The authors of this study declare that they do not have any conflict of interest.

Please refer to the accompanying ICMJE disclosure forms for further details.

Authors' contributions

S.W. designed and supervised the project; T.G., A.N., J.O. performed the experiments and analyzed the data with the help from S.L., Z.L, S-A-W., Y.L., C.Z., and S.W; S.W. and T.G. wrote the manuscript.

Data availability statement

RNA-seq dataset has been deposited to GEO (Gene Expression Omnibus). It will be publicly available once this manuscript is published.

Supplementary data

Supplementary data associated with this article can be found, in the online version, at https://doi.org/10.1016/j.jhepr.2024.101019.

References

Author names in bold designate shared co-first authorship

[1] Powell EE, Wong VW, Rinella M. Non-alcoholic fatty liver disease. Lancet 2021;397:2212–2224.

[2] Peiseler M, Schwabe R, Hampe J, et al. Immune mechanisms linking metabolic injury to inflammation and fibrosis in fatty liver disease - novel insights into cellular communication circuits. J Hepatol 2022;77:1136–1160.

[3] Hundertmark J, Berger H, Tacke F. Single cell RNA sequencing in NASH. Methods Mol Biol 2022;2455:181–202.

[4] Xiong X, Kuang H, Ansari S, et al. Landscape of intercellular crosstalk in healthy and NASH liver revealed by single-cell secretome gene analysis. Mol Cel 2019;75:644–660 e645.

[5] Wallace SJ, Tacke F, Schwabe RF, Henderson NC. Understanding the cellular interactome of non-alcoholic fatty liver disease. JHEP Rep 2022;4:100524.

[6] Fujita K, Nozaki Y, Wada K, et al. Effectiveness of antiplatelet drugs against experimental non-alcoholic fatty liver disease. Gut 2008;57:1583–1591.

[7] Dalbeni A, Castelli M, Zoncapè M, Minuz P, Sacerdoti D. Platelets in non-alcoholic fatty liver disease. Front Pharmacol 2022;13:842636.

[8] Malehmir M, Pfister D, Gallage S, et al. Platelet GPIbα is a mediator and potential interventional target for NASH and subsequent liver cancer. Nat Med 2019;25:641–655.

[9] Schwarzkopf K, Bojunga J, Rüschenbaum S, et al. Use of antiplatelet agents is inversely associated with liver fibrosis in patients with cardiovascular disease. Hepatol Commun 2018;2:1601–1609.

[10] Thongtan T, Deb A, Vutthikraivit W, et al. Antiplatelet therapy associated with lower prevalence of advanced liver fibrosis in non-alcoholic fatty liver disease: a systematic review and meta-analysis. Indian J Gastroenterol 2022;41:119–126.

[11] Campos GM, Bambha K, Vittinghoff E, et al. A clinical scoring system for predicting nonalcoholic steatohepatitis in morbidly obese patients. Hepatology (Baltimore, Md) 2008;47:1916–1923.

[12] Yoneda M, Fujii H, Sumida Y, et al. Platelet count for predicting fibrosis in nonalcoholic fatty liver disease. J Gastroenterol 2011;46:1300–1306.

[13] Milovanovic Alempijevic T, Stojkovic Lalosevic M, Dumic I, et al. Diagnostic accuracy of platelet count and platelet indices in noninvasive assessment of fibrosis in nonalcoholic fatty liver disease patients. Can J Gastroenterol Hepatol 2017;2017:6070135.

[14] Garjani A, Safaeiyan A, Khoshbaten M. Association between platelet count as a noninvasive marker and ultrasonographic grading in patients with nonalcoholic Fatty liver disease. Hepat Mon 2015;15:e24449.

[15] Shen H, Shahzad G, Jawairia M, Bostick RM, Mustacchia P. Association between aspirin use and the prevalence of nonalcoholic fatty liver disease: a cross-sectional study from the Third National Health and Nutrition Examination Survey. Aliment Pharmacol Ther 2014;40:1066–1073.

[16] Baenziger NL, Brodie GN, Majerus PW. Isolation and properties of a thrombin-sensitive protein of human platelets. J Biol Chem 1972;247:2723–2731.

[17] Li Y, Turpin CP, Wang S. Role of thrombospondin 1 in liver diseases. Hepatol Res 2017;47:186–193.

[18] Lopez-Dee Z, Pidcock K, Gutierrez LS. Thrombospondin-1: multiple paths to inflammation. Mediators Inflamm 2011;2011:296069.

[19] Li Y, Turpin CP, Wang S. Role of thrombospondin 1 in liver diseases. Hepatol Res 2017;47:186–193.

[20] Min-DeBartolo J, Schlerman F, Akare S, et al. Thrombospondin-I is a critical modulator in non-alcoholic steatohepatitis (NASH). PLoS One 2019;14:e0226854.

[21] Wang S. Thrombospondin1 deficiency attenuates obesity-associated microvascular complications in ApoE-/- mice. Sci Rep 2015;10:e0121403.

[22] Wang S, Lincoln TM, Murphy-Ullrich JE. Glucose downregulation of PKG-I protein mediates increased thrombospondin1-dependent TGF-{beta} activity in vascular smooth muscle cells. Am J Physiol Cel Physiol 2010;298:C1188–C1197.

[23] **Gwag T**, **Reddy Mooli RG**, **Li D**, et al. Macrophage-derived thrombospondin 1 promotes obesity-associated non-alcoholic fatty liver disease. JHEP Rep 2021;3:100193.

[24] Memetimin H, Li D, Tan K, et al. Myeloid-specific deletion of thrombospondin 1 protects against inflammation and insulin resistance in long-term diet-induced obese male mice. Am J Physiol Endocrinol Metab 2018;315:E1194–E1203.

[25] Virtue S, Vidal-Puig A. GTTs and ITTs in mice: simple tests, complex answers. Nat Metab 2021;3:883–886.

[26] Kim M, Yang SG, Kim JM, et al. Silymarin suppresses hepatic stellate cell activation in a dietary rat model of non-alcoholic steatohepatitis: analysis of isolated hepatic stellate cells. Int J Mol Med 2012;30:473–479.

[27] Liang W, Menke AL, Driessen A, et al. Establishment of a general NAFLD scoring system for rodent models and comparison to human liver pathology. PLoS One 2014;9:e115922.

[28] Kleiner DE, Brunt EM, Van Natta M, et al. Design and validation of a histological scoring system for nonalcoholic fatty liver disease. Hepatology (Baltimore, Md) 2005;41:1313–1321.

[29] Gwag T, Ma E, Zhou C, Wang S. Anti-CD47 antibody treatment attenuates liver inflammation and fibrosis in experimental non-alcoholic steatohepatitis models. Liver Int 2022;42:829–841.
[30] Folch J, Lees M, Sloane Stanley GH. A simple method for the isolation and purification of total lipides from animal tissues. J Biol Chem 1957;226:497–509.
[31] Sui Y, Xu J, Rios-Pilier J, Zhou C. Deficiency of PXR decreases atherosclerosis in apoE-deficient mice. J Lipid Res 2011;52:1652–1659.
[32] Xiang B, Zhang G, Ye S, et al. Characterization of a novel integrin binding protein, VPS33B, which is important for platelet activation and in vivo thrombosis and hemostasis. Circulation 2015;132:2334–2344.
[33] Clapper JR, Hendricks MD, Gu G, et al. Diet-induced mouse model of fatty liver disease and nonalcoholic steatohepatitis reflecting clinical disease progression and methods of assessment. Am J Physiol Gastrointest Liver Physiol 2013;305:G483–G495.
[34] Guo L, Zhang P, Chen Z, et al. Hepatic neuregulin 4 signaling defines an endocrine checkpoint for steatosis-to-NASH progression. J Clin Invest 2017;127:4449–4461.
[35] Wang GX, Zhao XY, Meng ZX, et al. The brown fat-enriched secreted factor Nrg4 preserves metabolic homeostasis through attenuation of hepatic lipogenesis. Nat Med 2014;20:1436–1443.
[36] Deuel TF, Senior RM, Chang D, et al. Platelet factor 4 is chemotactic for neutrophils and monocytes. Proc Natl Acad Sci U S A 1981;78:4584–4587.
[37] Kuijpers MJ, de Witt S, Nergiz-Unal R, et al. Supporting roles of platelet thrombospondin-1 and CD36 in thrombus formation on collagen. Arterioscler Thromb Vasc Biol 2014;34:1187–1192.
[38] Isenberg JS, Romeo MJ, Yu C, et al. Thrombospondin-1 stimulates platelet aggregation by blocking the antithrombotic activity of nitric oxide/cGMP signaling. Blood 2008;111:613–623.
[39] Prakash P, Kulkarni PP, Chauhan AK. Thrombospondin 1 requires von Willebrand factor to modulate arterial thrombosis in mice. Blood 2015;125:399–406.
[40] Aburima A, Berger M, Spurgeon BEJ, et al. Thrombospondin-1 promotes hemostasis through modulation of cAMP signaling in blood platelets. Blood 2021;137:678–689.
[41] Nagy Z, Vögtle T, Geer MJ, et al. The Gp1ba-Cre transgenic mouse: a new model to delineate platelet and leukocyte functions. Blood 2019;133:331–343.
[42] Ekinci I, Dumur S, Uzun H, et al. Thrombospondin 1 and nuclear factor kappa B signaling pathways in non-alcoholic fatty liver disease. J Gastrointestin Liver Dis 2022;31:309–316.
[43] Bronson SM, Westwood B, Cook KL, et al. Discrete correlation summation clustering reveals differential regulation of liver metabolism by thrombospondin-1 in low-fat and high-fat diet-fed mice. Metabolites 2022;12.
[44] Talme T, Bergdahl E, Sundqvist KG. Regulation of T-lymphocyte motility, adhesion and de-adhesion by a cell surface mechanism directed by low density lipoprotein receptor-related protein 1 and endogenous thrombospondin-1. Immunology 2014;142:176–192.
[45] Montenont E, Bhatlekar S, Jacob S, et al. CRISPR-edited megakaryocytes for rapid screening of platelet gene functions. Blood Adv 2021;5:2362–2374.
[46] Bhatlekar S, Basak I, Edelstein LC, et al. Anti-apoptotic BCL2L2 increases megakaryocyte proplatelet formation in cultures of human cord blood. Haematologica 2019;104:2075–2083.
[47] Bak RO, Dever DP, Porteus MH. CRISPR/Cas9 genome editing in human hematopoietic stem cells. Nat Protoc 2018;13:358–376.
[48] Villarroya F, Cereijo R, Villarroya J, Giralt M. Brown adipose tissue as a secretory organ. Nat Rev Endocrinol 2017;13:26–35.

Journal of Hepatology, Volume *6*

Supplemental information

Platelet-derived thrombospondin 1 promotes immune cell liver infiltration and exacerbates diet-induced steatohepatitis

Taesik Gwag, Sangderk Lee, Zhenyu Li, Alana Newcomb, Josephine Otuagomah, Steven A. Weinman, Ying Liang, Changcheng Zhou, and Shuxia Wang

Platelet-derived thrombospondin 1 promotes immune cell liver infiltration and exacerbates diet-induced steatohepatitis

Taesik Gwag, Sangderk Lee, Zhenyu Li, Alana Newcomb, Josephine Otuagomah,

Steven A. Weinman, Ying Liang, Changcheng Zhou, Shuxia Wang

Table of contents

Supplementary materials and methods……………………………………………………..……2

Supplementary references………………………………………………………,………,...4

Fig. S1…………………………………………………………………………………,……5

Fig. S2…………………………………………………………………………………,……6

Fig. S3…………………………………………………………………………………,……7

Fig. S4…………………………………………………………………………………,……8

Fig. S5…………………………………………………………………………………,……9

Table S1…………………………………………………………………………………...10

Table S2…………………………………………………………………………………...12

Table S3…………………………………………………………………………………...13

Whole western blot…………………………………………………………………………14

Supplementary materials and methods

Platelet preparation and aggregation

Blood was collected in ACD (85 mM trisodium citrate, 83 mM dextrose, and 21 mM citric acid) buffer. Platelets were isolated, washed, and resuspended in modified Tyrode's buffer (12 mM NaHCO3, 138 mM NaCl, 5.5 mM glucose, 2.9 mM KCl, 2 mM MgCl2, 0.42 mM NaH2PO4, 10 mM HEPES, pH 7.4). Platelet aggregation at 37 °C was measured by using a turbidimetric platelet aggregometer (Chrono-Log) with Collagen or Par4 (protease-activated receptor 4) agonist peptide (AYPGKF, from GenScript) stimulation as previously described (1, 2).

Mice bleeding time

Mice were anesthetized through inhalation of 2–5% isoflurane. Subsequently, the distal portion of the tail was cut and immersed in a warm saline solution. The time to stable cessation of bleeding was recorded as previous described (1).

Quantification of fat cell size and frequency distribution

H&E staining images from different fat depots' sections (eWAT, sWAT and BAT) were acquired. Adipocytes size was quantified by Image J and the frequency distribution was determined as previously described (3). Over 100 adipocytes were counted in each of 3 random sections per sample (n=5). We removed below size of 350 μm^2 from eWAT and sWAT and below size of 100 μm^2 from BAT to avoid including SVF (stromal vascular fraction) or non-adipocytes (4) (and a online eBook: Stock and Cinti, Encyclopedia of food sciences and Nutrition, 2003).

3T3-L1 (white preadipocyte cell line) and platelets co-culture, proliferation, and differentiation

Preadipocyte cell line, 3T3-L1 cells (ATCC), and wild-type or TSP1-deficient platelets ($4.5x10^4$/well, isolated from male 8-week-old mice) were co-cultured in a 24-well plate (with a

transwell insert) in growth medium (DMEM media with 5% FBS and 1% penicillin-streptomycin). The proliferation rate of 3T3-L1 cells was determined by a cell counter after 1, 3, 5, and 7 days of co-culture, and the growth curve was plotted. To determine whether platelet derived TSP1 affected 3T3 cell differentiation, confluent 3T3 cells were co-cultured with wild type or TSP1 deficient platelets ($4.5x10^4$/well) and treated with differentiation medium containing 0.5 mM IBMX, 1 μM dexamethasone, and 1 μM insulin in the growth media for 4-5 days (considering the short survival time of platelets in *in vitro* conditions). Lipid accumulation in differentiated adipocytes was determined by Oil red O staining. Expression of white adipocyte cell markers (AP2, C/EBPa, C/EBPb, PPAR-r, and adiponectin) were determined by qPCR.

T37i (brown preadipocyte cell line) differentiation, platelets co-culture, and Nrg4 production

T37i cells (provided by Dr. Jun Liu from Mayo Clinic) were cultured in growth medium (DMEM/F-12 containing 10% FBS, 1% penicillin-streptomycin, and 20 mM HEPES) in a 24-well plate. When cells reached full confluence, differentiation was induced by adding differentiation medium (growth medium containing 45 nM insulin and 10 μM T4) for 7 days. After differentiation, cells were cultured with wild-type or TSP1-deficient platelets (4.5x104/well) for 3 days. The Nrg4 or other gene expression level in cells was determined by qPCR, and the Nrg4 level in the conditioned medium was measured using the Nrg4 ELISA kit (Novus Biologicals, CO, USA).

Immunofluorescence staining of fat tissue with anti-CD41 antibody (maker for platelets)

To investigate platelet infiltration into fat tissues, paraffin sections from eWAT, sWAT, and BAT were stained with anti-CD41-PE (1:100, Thermofisher) overnight at 4°C. DAPI was used to stain nuclei. Images were captured by a Nikon Eclipse 55i microscope. Semi-quantification of CD41+ cells in liver sections was performed as previously described (5).

Statistical analysis

Statistical analysis was performed using Prism version 9.0 (GraphPad Software, San Diego, CA, USA). Data are expressed as mean values ± SE. Two-tailed Student's t-test was used to determine statistical significance between the two groups. One-way ANOVA followed by Tukey's multiple comparison test or 2-way ANOVA followed by Tukey's multiple comparison test was used for multi-group comparisons.

Supplementary references

1. Xiang B, Zhang G, Ye S, Zhang R, Huang C, Liu J, Tao M, et al. Characterization of a Novel Integrin Binding Protein, VPS33B, Which Is Important for Platelet Activation and In Vivo Thrombosis and Hemostasis. Circulation 2015;132:2334-2344.
2. Lu DH, Hsu CC, Huang SW, Tu HJ, Huang TF, Liou HC, Liao HM, et al. ARHGEF10 knockout inhibits platelet aggregation and protects mice from thrombus formation. J Thromb Haemost 2017;15:2053-2064.
3. Parlee SD, Lentz SI, Mori H, MacDougald OA. Quantifying size and number of adipocytes in adipose tissue. Methods Enzymol 2014;537:93-122.
4. Palomäki VA, Koivukangas V, Meriläinen S, Lehenkari P, Karttunen TJ. A Straightforward Method for Adipocyte Size and Count Analysis Using Open-source Software QuPath. Adipocyte 2022;11:99-107.
5. Gwag T, Ma E, Zhou C, Wang S. Anti-CD47 antibody treatment attenuates liver inflammation and fibrosis in experimental non-alcoholic steatohepatitis models. Liver Int 2022;42:829-841.

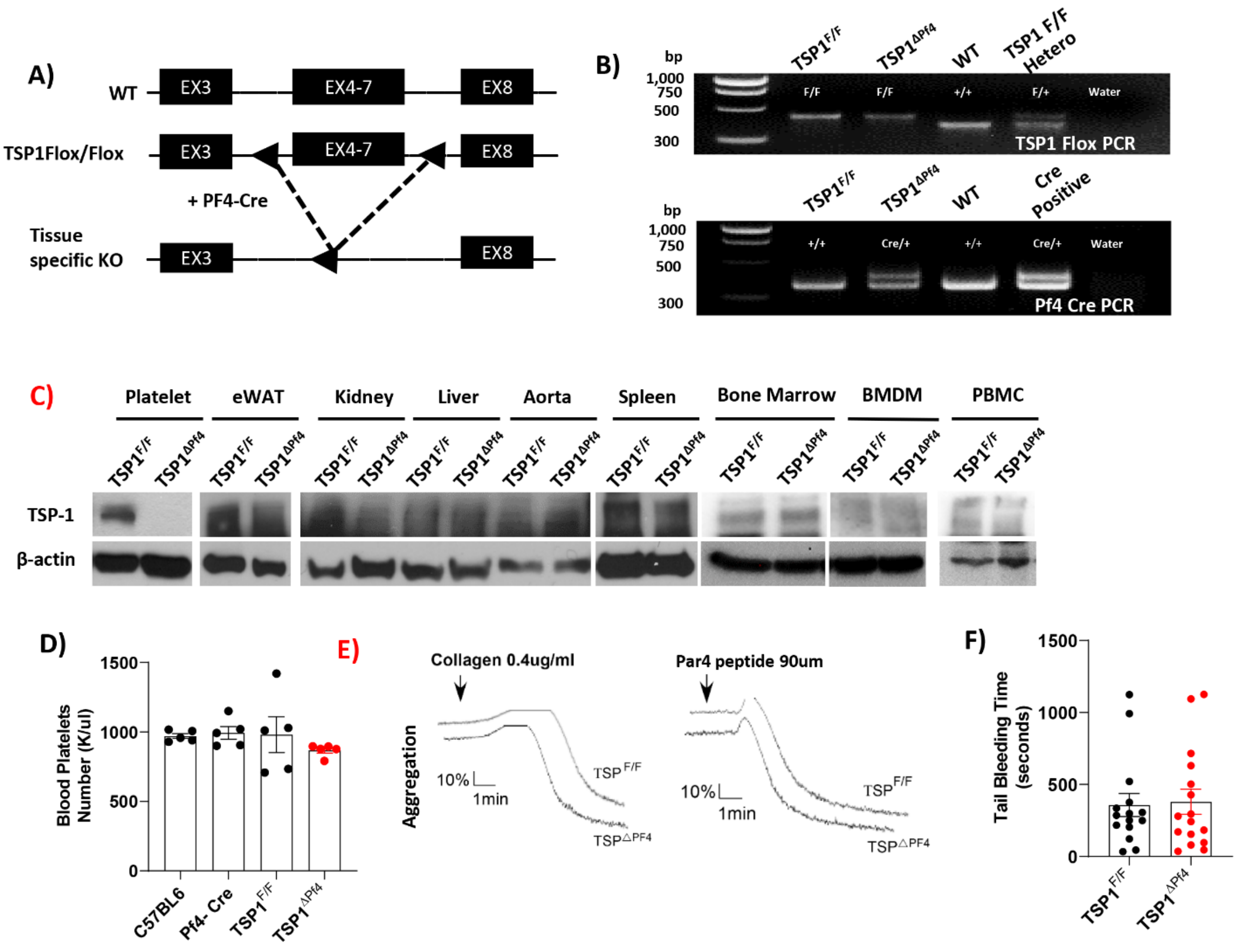

Fig. S1: Generation and characterization of mice with platelet specific deletion of TSP1 (TSP1$^{\Delta pf4}$).

A). Strategy for generation of TSP1$^{\Delta pf4}$ mice by breeding TSP1$^{F/F}$ mice with PF4-Cre mice; B) PCR analysis of genomic DNA from tail of wild type (WT), heterozygous, TSP1$^{F/F}$, or TSP1$^{\Delta pf4}$ mice from 8 week old male mice for detection of floxed gene and Cre gene, respectively; C) Expression of TSP1 from isolated platelets, various tissues or blood immune cells from 8 weeks old male mice was determined by immunoblotting; D) Blood platelet number; E) Platelet aggregation measured in a lumi-Aggregometer with Collagen or Par4 peptide stimulation and F) Mice tail bleeding time were measured. Results are mean ± SE (n=5 or 16 mice/group). eWAT: white adipose tissue (from epididymal fat); PLT: platelet; BMDM: bone marrow derived macrophages; PBMC: peripheral blood mononuclear cells; Par4: protease-activated receptor 4

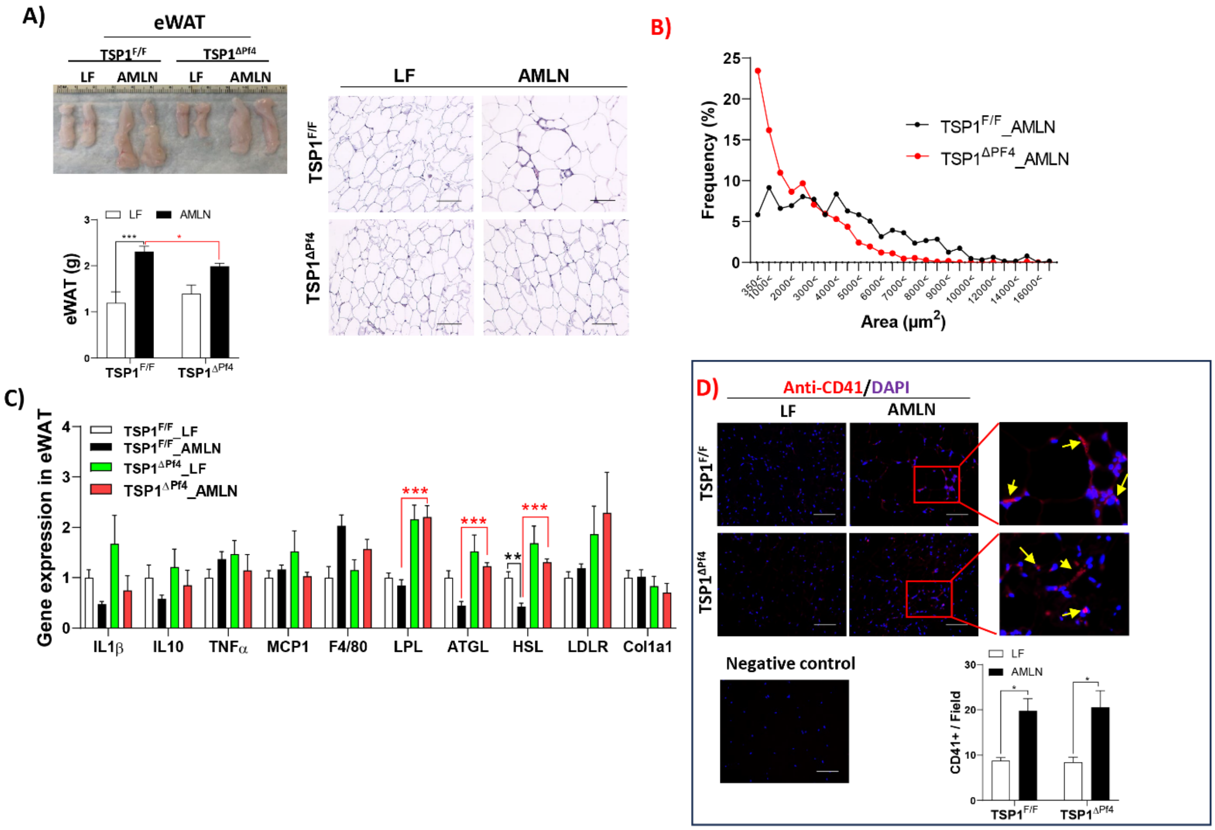

Fig. S2: Changes in epididymal white fat tissue from TSP1$^{\Delta pf4}$ mice after 32 weeks of AMLN diet feeding

(A). Weight and representative H&E staining images of epididymal fat (eWAT) from 32 weeks of LF or AMLN fed TSP1 $^{F/F}$ mice or TSP1$^{\Delta pf4}$ mice; (B). The distribution of adipocyte sizes; (C) qPCR of gene expression; Data are represented as mean ± SE (n=3-5 mice/group); 2-way ANOVA with Tukey's multiple comparisons test. *P<0.05 and *** P<0.001; (D). Representative fluorescence images of eWAT staining with anti-CD41 antibody (a platelet marker). Positive staining was shown as yellow arrows.

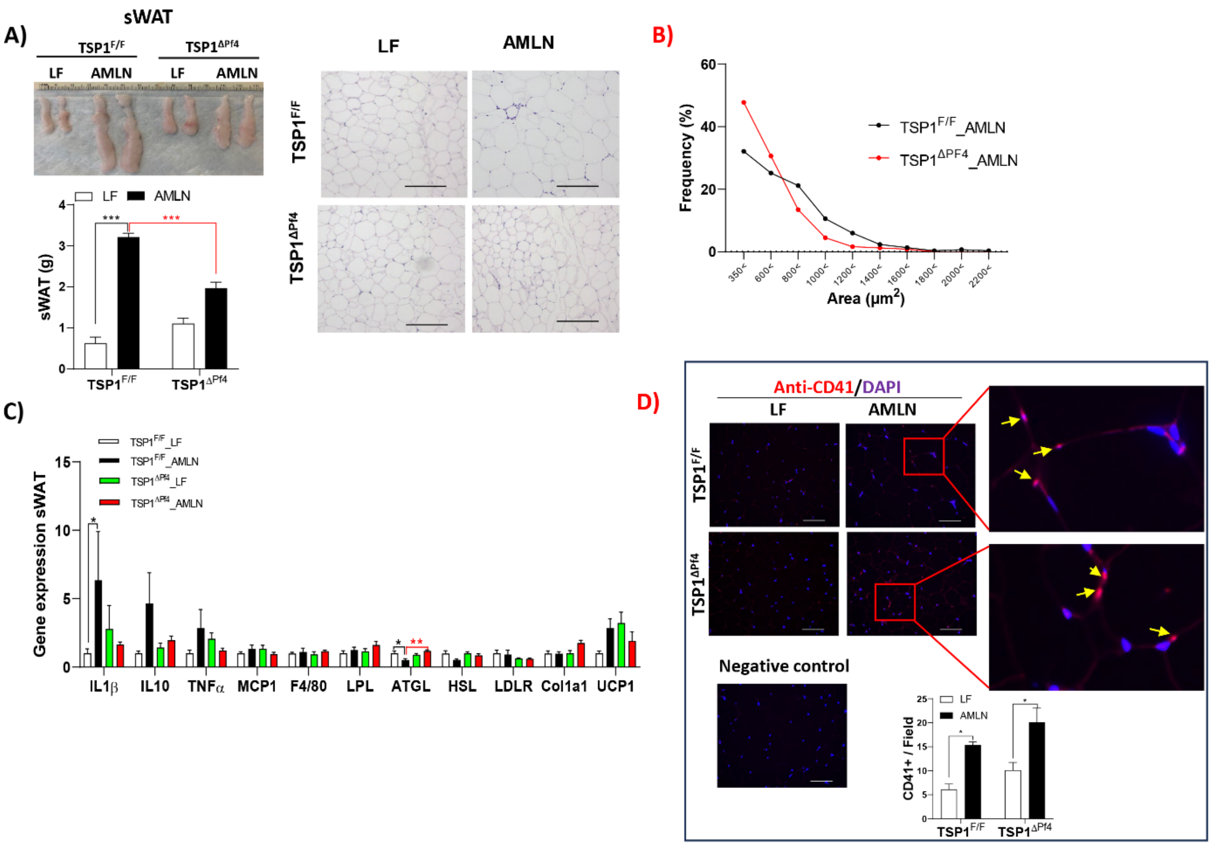

Fig. S3: Changes in subcutaneous white fat tissue from TSP1$^{\Delta pf4}$ mice after 32 weeks of AMLN diet feeding

(A). Weight and representative H&E staining images of subcutaneous fat (sWAT) from 32 weeks of LF or AMLN fed TSP1$^{F/F}$ mice or TSP1$^{\Delta pf4}$ mice; (B). The distribution of adipocyte sizes; (C) qPCR of gene expression. Data are represented as mean ± SE (n=3-5 mice/group); 2-way ANOVA with Tukey's multiple comparisons test. *P<0.05, ** P<0.01 and *** P<0.001; (D). Representative fluorescence images of sWAT staining with anti-CD41 antibody (a platelet marker). Positive staining was shown as yellow arrows.

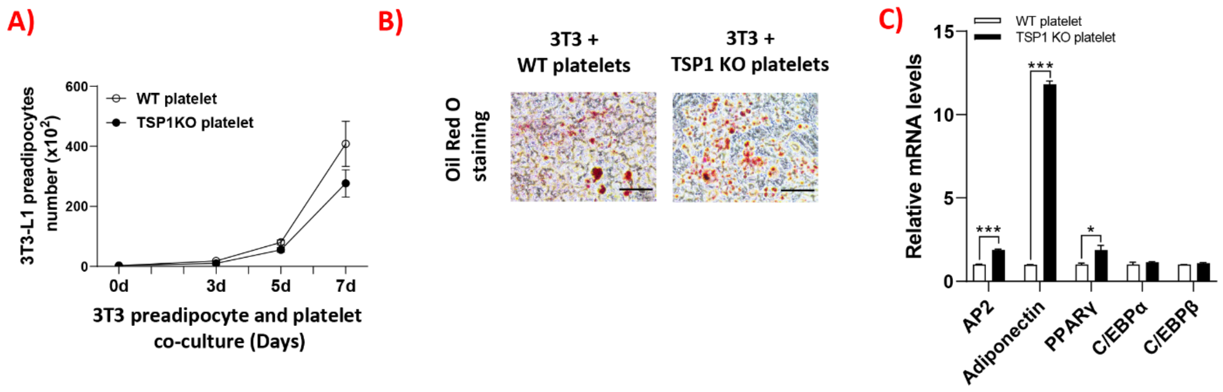

Fig. S4: Co-culture of platelets with 3T3-L1 preadipocytes on 3T3 preadipocyte proliferation and differentiation

(A). Proliferation curve of 3T3-L1 preadipocytes after various days of co-culturing with WT or TSP1KO platelets. Differentiation was induced in co-cultured 3T3-L1 cell and platelets. Representative Oil Red O staining (B) and qPCR of gene expression in differentiated 3T3 cells (C) were performed. Data are represented as mean ± SE (n=3); 2-way ANOVA with Tukey's multiple comparisons test. *P<0.05 and *** P<0.001

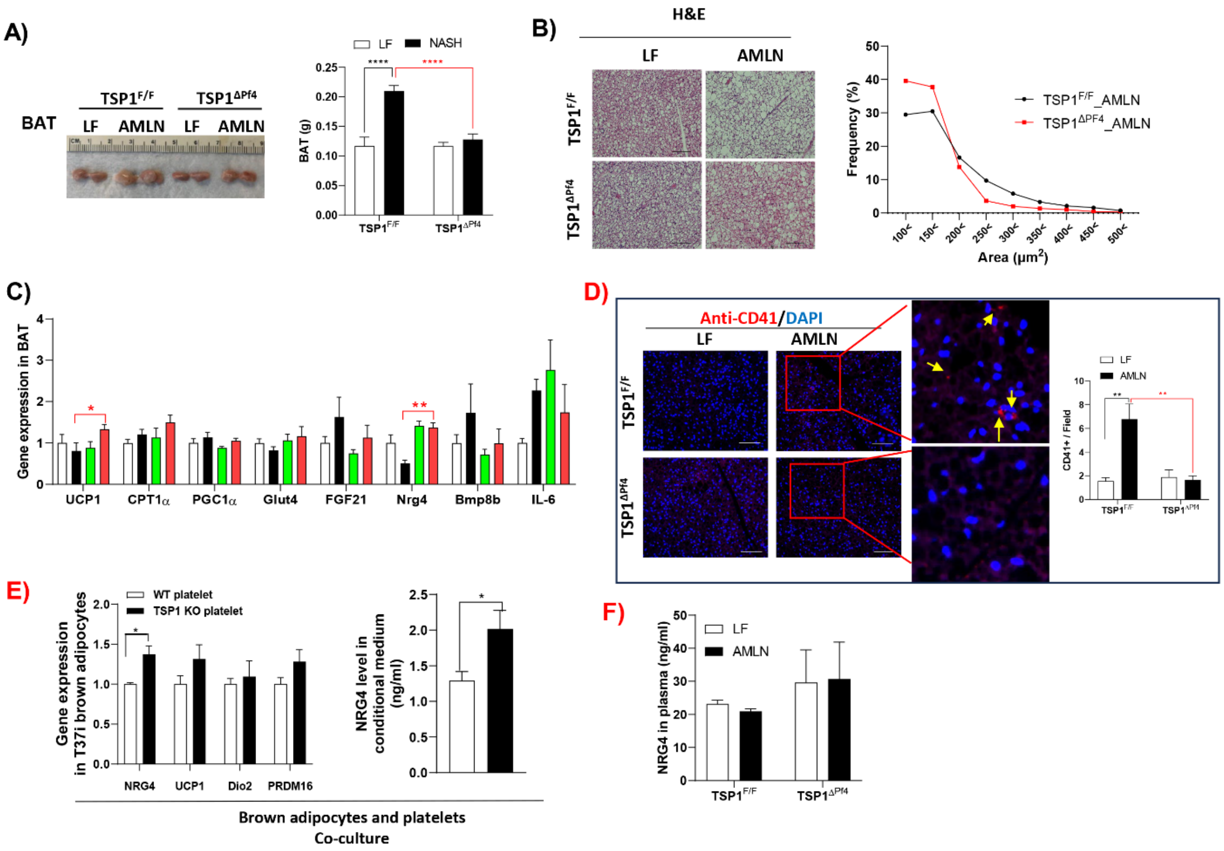

Fig. S5: Changes in brown fat tissue from *TSP1^Δpf4^* mice after 32 weeks of AMLN diet feeding and in vitro platelets and brown adipocytes co-culture

(A). Weight and representative H&E staining images of BAT from 32 weeks of LF or AMLN fed $TSP1^{F/F}$ mice or $TSP1^{\Delta pf4}$ mice; (B). The distribution of adipocyte sizes; (C) qPCR of gene expression; (D). Representative fluorescence images of BAT staining with anti-CD41 antibody (a platelet marker). Positive staining was shown as yellow arrows; (E). qPCR of gene expression or Nrg4 protein levels in condition media in T37i brown adipocytes after co-cultured with WT or TSP1KO platelets for 3 days (n=3 experiments); (F). Plasma Nrg4 protein levels from 32 weeks of LF or AMLN fed *$TSP1^{F/F}$* mice or *$TSP1^{\Delta pf4}$* mice. Data are represented as mean ± SE (n=3-5 mice/group); 2-way ANOVA with Tukey's multiple comparisons test. *P<0.05, ** P<0.01 and **** P<0.0001

Table S1. Primer Sequences for qPCR

Gene	Primer sequence	Genes	Primer sequence
		**Mouse primers**	
CPT1α	5'-CTCTATGTGGTGTCCAAG-3'	PGC1α	5'-CTGCATGAGTGTGTGCTGTG-3'
	5'-CACAGGACACATAGTCAG-3'		5'-CAAATATGTTCGCAGGCTCA-3'
Glut4	5'-CATGGCTGTCGCTGGTTTC-3'	FGF21	5'-GCTGCTGGAGGACGGTTACA-3'
	5'-AAACCCATGCCGACAATGA-3'		5'-CACAGGTCCCCAGGATGTTG-3'
Nrg4	5'-ATGCCAACAGATCACGAGC-3'	Bmp8b	5'-CAACCACGCCACTATGCAG-3'
	5'-TCTTCAGTGTTCTCTGTGGCTG-3'		5'-CACTCAGCTCAGTAGGCACA-3'
F4/80	5'-CTTTGGCTATGGGCTTCCAGTC-3'	CD11b	5'-CGGAAAGTAGTGAGAGAACTGTTTC-3'
	5'-GCAAGGAGGACAGAGTTTATCGTG-3'		5'-TTATAATCCAAGGGATCACCGAATTT-3'
UCP1	5'-ACTGCCACACCTCCAGTCATT-3'	MCP-1	5'-CAGCCAGATGCAGTTAACGC-3'
	5'-CTTTGCCTCACTCAGGATTGG-3'	(CCl2)	5'-GCCTACTCATTGGGATCATCTTG-3'
IL-1β	5'-TGGAGAGTGTGGATCCCAAGCAAT-3'	TNFα	5'-AGCCGATGGGTTGTACCT-3'
	5'-TGTCCTGACCACTGTTGTTTCCCA-3'		5'-TGAGTTGGTCCCCCTTCT-3'
TGFβ	5'-ACAATTCCTGGCGTTACC-3'	α-SMA	5'-ATTGTGCTGGACTCTGGAGATGGT-3'
	5'-GGCTGATCCCGTTGATTT-3'		5'-TGAGTCACGGACAATCTCACGCT-3'
TIMP1	5'-TCTTGGTTCCCTGGCGTACTCT-3'	Col1a1	5'-TTCTCCTGGCAAAGACGGACTCAA-3'
	5'-GTGAGTGTCACTCTCCAGTTTGC-3'		5'-AGGAAGCTGAAGTCATAACCGCCA-3'
IL-6	5'-TGGCTAAGGACCAAGACCATCCAA-3'	CD68	5'- CAAGGTCCAGGGAGGTTGTG -3'
	5'-AACGCACTAGGTTTGCCGAGTAGA-3'		5'- CCAAAGGTAAGCTGTCCATAAGGA -3'
Clec4f	5'-CTTCGGGGAAGCAACAACTC-3'	CXCL1	5'- TAGTAGAAGGGTGTTGTG-3'
	5'-CAAGCAACTGCACCAGAGAAC-3'		5'- GTAACAGTCCTTTGAACG-3'
CXCL2	5'-CTG TCT GAG AGT TCA CTT A-3'	CXCL4	5'- CCCTAGACCCATTTCCTCAA-3'
	5'-GTA GCT AGT TCC CAA CTC-3'		5'- AGAAACAACAGGCCCAGAAG-3'
CXCL5	5'-ACAGTGCCCTACGGTGGAAGT-3'	CXCL7	5'-GGAAAATCTGATGGCATGGAC-3'
	5'-CGAGTGCATTCCGCTTAGCTT-3'		5'-CAGGCACGTTTTTTGTCCATTCT-3'
CXCL10	5'-CCTCATCCTGCTGGGTCTG-3'	CXCL12	5'-TGCATCAGTGACGGTAAACCA-3'
	5'-CTCAACACGTGGGCAGGA-3'		5'-AGATGCTTGACGTTGGCTCT-3'
CXCR2	5'-TCACAAACAGCGTCGTAGA-3'	CXCR4	5'-TCAGTGGCTGACCTCCTCTT-3'
	5'-GACAGCATCTGGCAGAATAG-3'		5'-CTTGGCCTTTGACTGTTGGT-3'

IL10	5'- GCTCTTACTGACTGGCATGAG- 3'<br>5'- CGCAGCTCTAGGAGCATGTG -3'	CCR2	5’-AGAGAGCTGCAGCAAAAAGG-3’<br>5’-GGAAAGAGGCAGTTGCAAAG-3’
ATGL	5'- AACACCAGCATCCAGTTCAA-3'<br>5'- GGTTCAGTAGGCCATTCCTC-3'	LPL	5’-GGGAGTTTGGCTCCAGAGTTT-3’<br>5’-TGTGTCTTCAGGGGTCCTTAG-3’
LDLR	5'-GCTCCATAGGCTATCTGCTCTTCA-3'<br>5'-GCGGTCCAGGGTCATCTTC-3'	HSL	5’-GGCTCACAGTTACCATCTCACC-3’<br>5’-GAGTACCTTGCTGTCCTGTCC-3’
AP2	5'-AAGCCCACTCCCACTTCTTT-3'<br>5'-TCACCTGGAAGACAGCTCCT-3'	Dio2	5'-CATCTTCCTCCTAGATGCCTA-3'<br>5'-CTGATTCAGGATTGGAGACGTG-3'
Adiponectin	5'-AACATTCCGGGACTCTACT-3'<br>5'-TACTGGTCGTAGGTGAAGAG-3'	PRDM16	5'-CACAAGACATCTGAGGACAC-3'<br>5'-CTCGTGTTCGTGCTTCTT-3'
PPARγ	5'-TGCTGTTATGGGTGAAACTCTG-3'<br>5'-CTGTGTCAACCATGGTAATTTCTT-3'	C/EBPα	5'-TGCCTCCCAGAGGACCAATGAAAT-3'<br>5'-TGTGTGTATGAACTGGCTGGAGGT-3'
C/EBPβ	5'-TGATGCAATCCGGATCAAACGTGG-3'<br>5'-TTTAAGTGATTACTCAGGGCCCGGCT-3'		
**Human primers**			
α-SMA	5’-CCAGAGCCATTGTCACACAC-3’<br>5’-CAGCCAAGCACTGTCAGG-3’	IL-1β	5’-CAACAGGCTGCTCTGGGATT-3’<br>5’-CATGGCCACAACAACTGACG-3’
TIMP	5’-GGAGAGTGTCTGCGGATACTTC-3’<br>5’-GCAGGTAGTGATGTGCAAGAGTC-3’	β-Actin	5’-CATGTACGTTGCTATCCAGGC -3’<br>5’-CTCCTTAATGTCACGCACGAT -3’

Table S2. Blood profile for 8 weeks-old mice

Parameter	$TSP^{F/F}$	$TSP1^{\Delta pf4}$
WBC count (k/ul)	4.24±0.754	4.24±0.545
Neutrophil count (K/ul)	0.73±0.245	1.11±0.591
Lymphocytes count (K/ul)	3.36±0.529	2.89±0.666
Monocytes count (K/ul)	0.06±0.024	0.05±0.025
Eosinophil count (K/ul)	0.09±0.027	0.05±0.025
RBC (M/ul)	9.59±0.469	9.20±0.173
Hemoglobin (g/dL)	14.32±0.602	13.78±0.286
MCV (fL)	50.68±0.712	50.84±0.631
MCH (Pg)	14.94±0.195	14.98±0.311
MCHC (g/dL)	29.48±0.402	29.44±0.241
RDW (%)	23.36±0.404	22.24±0.522
Platelet (K/ul)	980.4±287.863	868±43.537

Abbreviations: RBC: red blood cell; MCV: mean corpuscular volume; MCH: Mean corpuscular hemoglobin; MCHC: mean corpuscular hemoglobin concentration; RDW: red cell distribution width; MPV: mean platelet volume

Table S3. Blood profile in mice after 32 weeks AMLN or LF diet feeding

Parameter	$TSP1^{F/F}$_LF	$TSP1^{F/F}$_AMLN	$TSP1^{\Delta pf4}$_LF	$TSP1^{\Delta pf4}$_AMLN
WBC count (k/ul)	5.26±1.265	8.19±3.875 *	5.25±1.911	4.95±1.088 ##
Neutrophil count (K/ul)	1.99±1.157	1.53±0.402	1.93±1.187	1.75±0.958
Lymphocytes count (K/ul)	3.12±1.191	6.20±3.618 ***	3.02±1.512	3.04±1.324 ###
Monocytes count (K/ul)	0.09±0.072	0.23±0.075 **	0.07±0.036	0.13±0.194 ##
Eosinophil count (K/ul)	0.08±0.049	0.14±0.061	0.09±0.046	0.10±0.050
RBC (M/ul)	10.02±0.438	10.28±1.563	10.04±0.707	9.95±0.995
Hemoglobin (g/dL)	14.74±0.872	14.60±2.250	14.49±1.138	14.27±1.223
MCV (fL)	49.50±1.920	47.89±1.176 *	48.17±1.040	47.74±0.632
MCH (Pg)	14.64±0.346	14.19±0.398*	14.43±0.369	14.30±0.286
MCHC (g/dL)	29.84±0.536	29.65±0.655	29.95±0.538	30.12±0.960
RDW (%)	25.22±1.364	26.03±1.584	29.61±1.189	30.63±2.302
Platelet (K/ul)	987±422.570	1097±146.007	1254±153.848	1258±262.188

Note: ****P*<0.05, *****P***<0.01, and ******P***<0.001 as compared to $TSP1^{F/F}$_LF;**

#*P*<0.05 and ## *P*<0.01 as compared to $TSP1^{F/F}$_AMLN

Abbreviations: RBC: red blood cell; MCV: mean corpuscular volume; MCH: Mean corpuscular hemoglobin; MCHC: mean corpuscular hemoglobin concentration; RDW: red cell distribution width; MPV: mean platelet volume

Whole western blot for Figure 3G, Figure 4B

Fig. 3G

α-SMA in liver

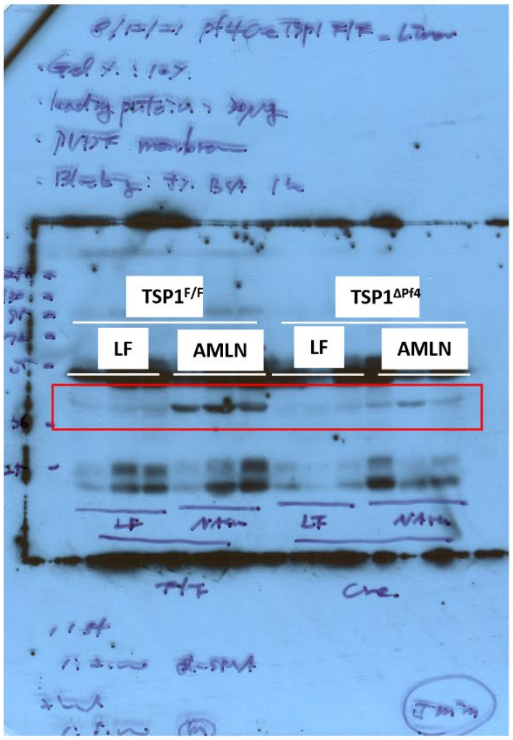

β-actin for α-SMA

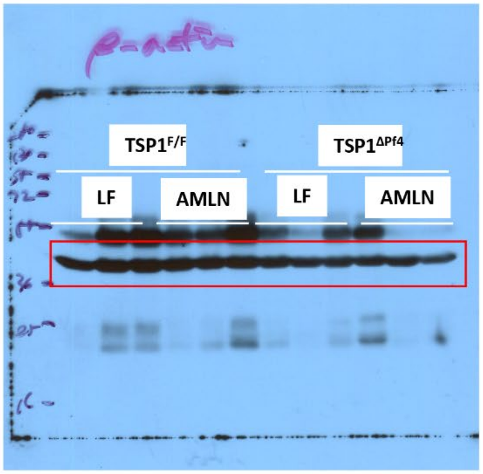

Fig4. B

TSP1 in liver (reducing gel)

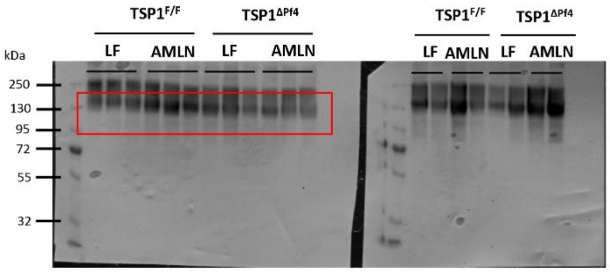

β-actin for TSP1 in liver

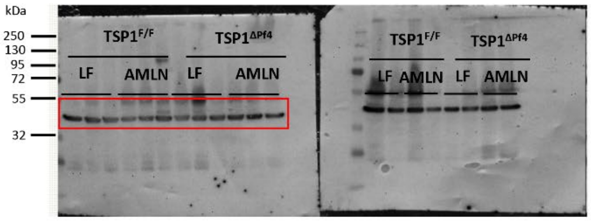

Whole western blot for Figure S1

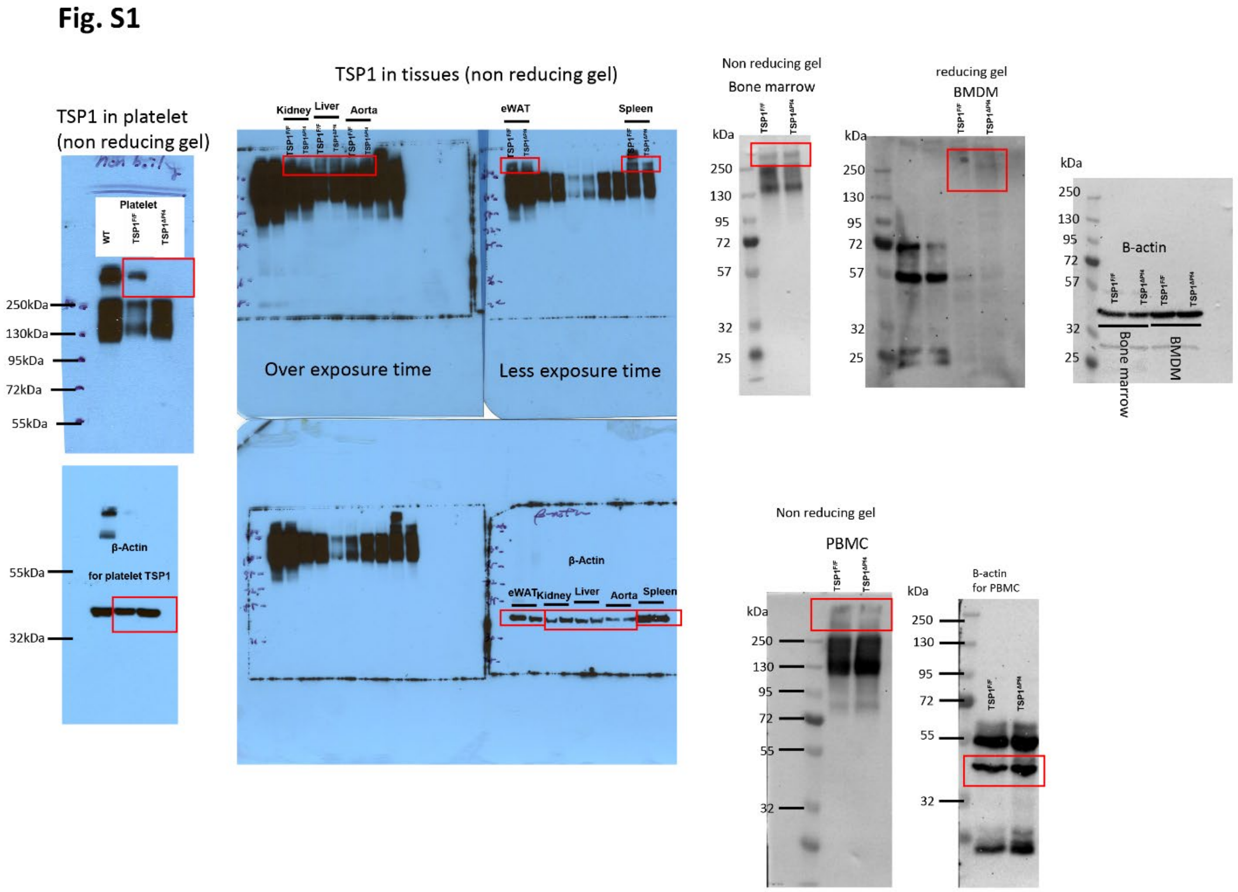

Supplement: Multimedia component 4 [file mmc4.pdf]
